# Anisotropic fracture mechanisms and crack-mediated mechanical degradation in trigraphene nanosheets: A molecular dynamics study

**DOI:** 10.1371/journal.pone.0354703

**Published:** 2026-08-26

**Authors:** Yuan Cheng, Yanting Sun

**Affiliations:** 1 School of Control Engineering, Wuxi University of Technology, Wuxi, Jiangsu, China; 2 College of Educational Sciences, Jiangsu Second Normal University, Nanjing, Jiangsu, China; ICFAI Foundation for Higher Education Faculty of Science and Technology, INDIA

## Abstract

This study investigates the anisotropic fracture mechanisms and mechanical degradation of trigraphene nanosheets (TNS) with central cracks using molecular dynamics (MD) simulations. The elastic modulus, ultimate stress, fracture strain, toughness, and stress intensity factor (${\text{K}}_{\text{c}}$) are systematically evaluated as functions of crack angle (0°–90°), crack length (30–60 Å), and temperature (200–1000 K) under uniaxial tension along armchair (X) and zigzag (Y) directions. Key findings reveal direction-dependent failure modes: X-loading causes brittle, mode-I fracture with symmetric crack propagation and high stress concentration at tips, while Y-loading induces crack deflection toward boundaries due to diffuse stress distribution and tip shielding. Mechanically, the armchair (X) direction exhibits higher elastic modulus, whereas zigzag (Y) demonstrates superior ultimate stress, fracture strain, toughness, and fracture toughness but greater sensitivity to crack orientation. Elevated temperatures lead to significant thermal softening, reducing stiffness, strength, and fracture resistance, with the Y direction experiencing more pronounced deterioration.

## 1. Introduction

Two-dimensional (2D) carbon allotropes represent a class of materials consisting exclusively of carbon atoms arranged in distinct planar configurations. These structures exhibit a broad spectrum of physical and electronic properties due to variations in their atomic bonding networks, rendering them highly adaptable for diverse technological applications. The exploration of novel 2D carbon allotropes is motivated by the need to address inherent limitations of conventional materials such as graphene, most notably its zero bandgap, while expanding their utility in next-generation technologies, including nanoelectronics, nanocomposite, energy storage, and photoconversion systems [1,2]. Recent advances have led to the discovery of numerous 2D carbon allotropes, including planar net-τ [3], net C, net W, net Y [4], graphenylene [5], S-graphene [6], Ψ-graphene [7], PBCF graphene (P6/mmm space group with 24 carbon atoms in a hexagonal unit cell denoted as PBCF-graphene) [8], penta-graphene [9], PHOTH-graphene [10], a novel 2D planar carbon allotrope formed by 4-5-6-7-8 carbon rings (hence the name: Pentagon, Hexagon, Octagon, Tetragon, Heptagon), Dodecanophene [11], γ-graphyne [12], R12 graphene [13], THD-graphene (all-*sp*^2^ hybridized carbon sheet with tetragon, hexagon and dodecagon rings named as THD-graphene) [14], HOP-graphene (Hexagon-Octagon-Pentagon graphene) [15], Spiro graphene [16], TODD-graphene [17], an innovative 2D planar carbon allotrope with a distinctive porous arrangement comprising 3-8-10-12 carbon rings (hence the name: Triangle, Octagon, Decagon, Dodecagon) and trigraphene [18], among others. These materials represent a structurally diverse and functionally versatile family with significant potential in nanotechnology, electronics, and energy storage. Their unique properties highlight their importance in advancing materials science. The continued exploration of novel carbon allotropes may yield materials with unprecedented mechanical, electronic, and thermal properties, enabling breakthroughs in flexible electronics, energy storage, and biomedical devices for next-generation technologies.

Trigraphene nanosheets (TNS) represent a novel carbon allotrope that has garnered significant interest due to their unique structural properties and potential applications in various fields. Structurally, trigraphene is characterized by the substitution of sp² carbon atoms in graphene with carbon trigon configurations, giving rise to a distinct arrangement of carbon atoms. This type of structural modification results in materials that are both porous and exhibit intriguing physical attributes. Trigraphene and its derivatives are metastable yet dynamically stable, suggesting that they can maintain structural integrity under various conditions while showcasing significant potential for real-world applications [18]. The electronic properties of trigraphene nanosheets contribute significantly to their applicability in electronic devices. In particular, studies have shown that trigraphene possesses metallic properties, rendering it suitable for conductive applications. Additionally, associated structures like g-trigraphyne have been classified as indirect band gap semiconductors, affirming the diversity in electronic characteristics among these materials [19]. Chen et al. [20] confirmed the metallic nature and high conductivity of TNS. Upon functionalization with lithium (Li), sodium (Na), potassium (K), and calcium (Ca), H_2_ adsorption studies revealed Li@ TNS, Na@ TNS, and Ca@ TNS achieved stable storage capacities of 24, 14, and 24 H_2_ molecules (13.99 wt%, 8.26 wt%, and 12.77 wt%, respectively), whereas pristine TNS and K@ TNS showed negligible adsorption. Mondal and Datta [21] investigated negative thermal expansion (NTE) in 2D carbon materials. TNS exhibits exceptionally large NTE, persisting up to 4200 K due to soft phonon modes that drive rotational distortions in its 3-membered rings, as confirmed by ab initio simulations. This behavior mirrors rigid-unit modes observed in bulk systems. TNS represent a metastable 2D carbon allotrope with demonstrated potential in nanoelectronics and energy storage [18–21]. While their electronic properties (e.g., metallic conductivity [20]) and anomalous thermal expansion [21] have been studied, their mechanical behavior—particularly under defects, remains unexplored. This gap is critical given that nanostructured materials exhibit size-dependent properties inaccessible to classical continuum models. Recent advances in nonlocal elasticity (e.g., mixture unified gradient theory [22] and stress-driven formulations [23]) resolve such scale effects but require atomistic validation. A fundamental challenge in multiscale modeling of fracture arises from strain softening, which renders the boundary value problem ill-posed at the macroscopic scale [22]. As discussed by Talebi et al. [24], standard homogenization techniques such as FE² lose their validity when strain localization occurs, because the representative volume element (RVE) ceases to be representative when deformation localizes into narrow bands. To address this issue, specialized multiscale methods for fracture have been developed, including concurrent coupling techniques that directly embed fine-scale models (molecular dynamics or finely discretized continuum) in regions where fracture processes occur, while using coarse-scale continuum models elsewhere. The PERMIX framework [24] represents a significant advancement in this direction, providing an open-source platform that couples XFEM for continuum fracture with molecular dynamics (LAMMPS) through the Arlequin/bridging domain method. This approach allows accurate resolution of crack tip processes while maintaining computational efficiency in regions far from the crack.

The present study contributes to this multiscale vision by establishing the first comprehensive database of mechanical properties and fracture parameters for defective trigraphene at the atomistic scale. These data, including elastic modulus, ultimate strength, fracture toughness ($K_{c}$), and critical energy release rate ($G_{c}$) as functions of crack geometry and temperature, provide essential input parameters for higher-scale models. For instance, the cohesive zone properties required in concurrent multiscale frameworks [24] can be derived from the traction-separation relationships implicit in our stress-strain curves. Similarly, the anisotropic fracture mechanisms we observe (mode-I brittle fracture vs. crack deflection) inform the choice of enrichment functions and crack propagation criteria in continuum XFEM models. Future work should integrate these atomistic results into multiscale frameworks such as PERMIX to enable device-scale simulations of trigraphene-based structures with ab initio-informed accuracy. Here, we bridge this divide by employing NEMD simulations to quantify fracture anisotropy and crack-mediated degradation in TNS. Our work provides: (i) the first mechanical property database for defective TNS, (ii) atomistic insights into failure mechanisms tied to its trigonal topology, and (iii) critical benchmarks for calibrating nonlocal continuum theories [25,26].

Defects are inherently present in fabricated nanomaterials and significantly influence their mechanical properties. In nanosheets, imperfections such as notches and cracks act as stress concentrators, degrading mechanical performance by facilitating localized failure [27,28]. A thorough understanding of these defect-mediated mechanisms is crucial for optimizing material design and ensuring structural reliability. While nanosheets possess exceptional intrinsic mechanical properties, their practical performance is highly sensitive to defect presence, necessitating defect-aware engineering approaches. For instance, Wang et al. [29] identified that electrocatalytic activation takes place preferentially at line defects in layered double hydroxide nanosheets, leading to nonisotropic compressive strain and potential structural failures due to mechanical forces generated along these defects. Specifically, not every defect leads to cracking; rather, the geometrical arrangement and types of these defects govern the mechanical outcome [29]. Bao et al. analyzed crack propagation in MoS2 nanosheets, demonstrating that localized brittle failures can occur at defect sites under strain [30]. Defects like notches and line cracks create localized stress concentrations, leading to a decrease in the material’s ability to withstand loads. The stress concentration factor depends on the defect geometry, size, and orientation [31,32]. Notches and line cracks lead to brittle fracture behavior, especially under tensile loading [33]. The mechanical properties of graphene exhibit significant dependence on both crack orientation, distinguished between zigzag and armchair configurations, and applied strain rate [34].

While prior studies have elucidated the electronic, thermal, and adsorption properties of TNS, their mechanical behavior, particularly under defect-induced stress, remains unexplored. This work addresses this critical gap by systematically quantifying the fracture mechanics and anisotropic mechanical properties (elastic modulus, ultimate strength, toughness, and stress intensity factor) of defective TNS with pre-existing cracks, using non-equilibrium molecular dynamics (NEMD) simulations. For the first time, we reveal: (1) the direction-dependent crack propagation mechanisms (brittle mode-I vs. deflected failure) governed by TNS’s unique trigonal topology; and (2) the coupled effects of crack geometry (length/orientation) and temperature (200–1000 K) on mechanical degradation; and its fracture resistance. These insights establish a foundational understanding of TNS’s mechanical reliability for nanoelectronic and energy storage applications, where structural defects are inevitable.

## 2. Simulation detail

**Fig 1:**
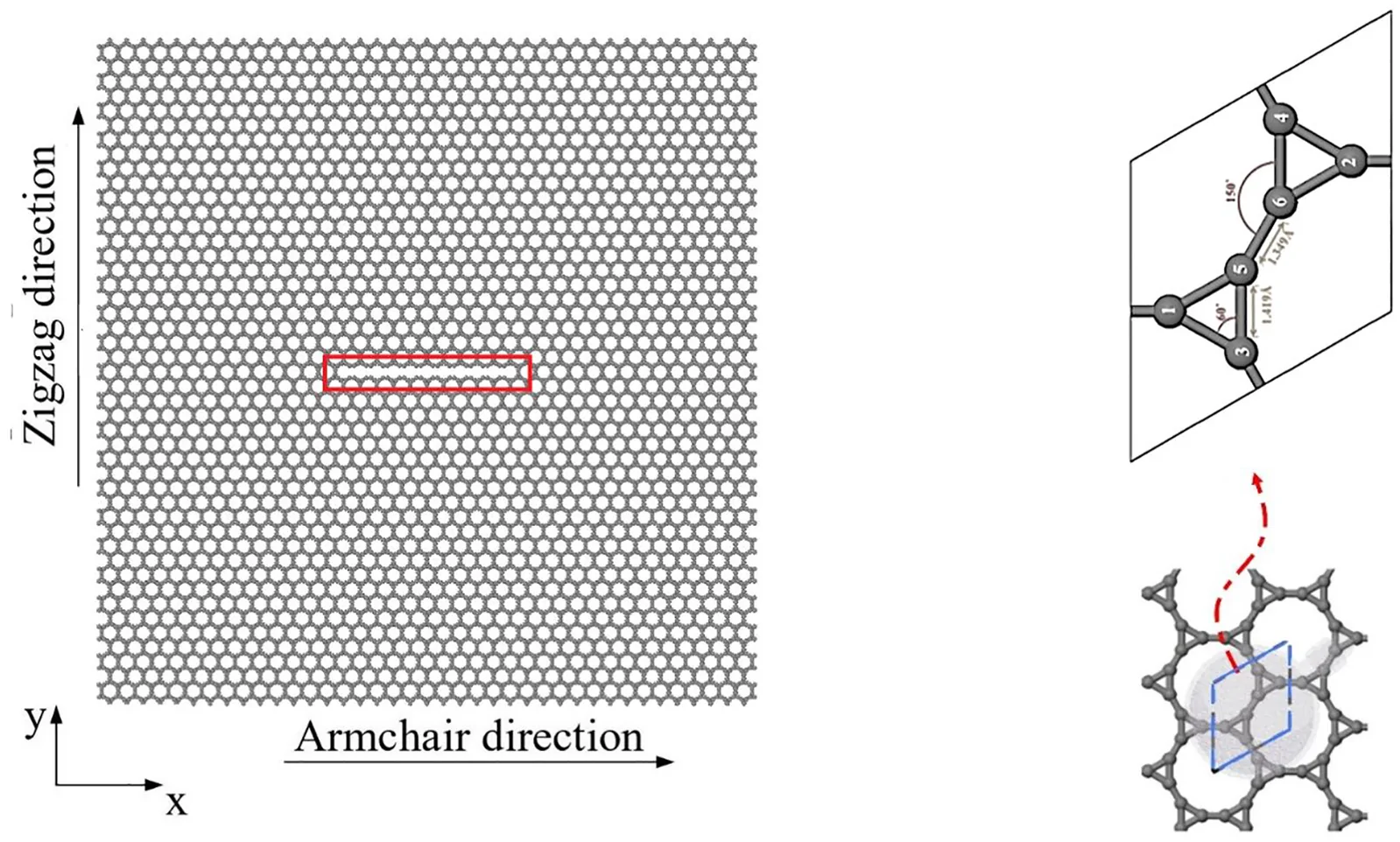
Schematic representation of a TNS with pre-existing line crack.

The structural configuration of a TNS containing a pre-existing linear crack is illustrated in Fig 1. The crack angle (θ), defined as the angle between the crack line and loading axis, is varied from 0° to 90°.This novel two-dimensional material was synthesized by systematically replacing each carbon atom in conventional graphene with a carbon trigon, resulting in a unit cell comprising six symmetry-equivalent sp²-hybridized carbon atoms arranged in a hexagonal lattice (a=b=5.19 Å). Detailed structural characterization reveals two distinct bonding configurations: (1) intra-trigon C-C bonds measuring 1.419 Å and (2) inter-trigon separations of 1.349 Å. Notably, the system exhibits significant angular distortions from ideal sp² hybridization, with characteristic bond angles of 60° (C1-C3-C4) and 150° (C4-C6-C5) compared to graphene’s uniform 120° configuration. These structural anomalies suggest the presence of substantial lattice strain, which may contribute to the material’s enhanced cohesive energy properties [18].

The mechanical properties and fracture behavior of TNS are investigated using classical reactive molecular dynamics simulations with fully atomistic models implemented in LAMMPS [34,35]. The AIREBO-M [36] potential is selected for its proven accuracy in modeling carbon-carbon bond breaking and formation in 2D materials, incorporating both Lennard-Jones (long-range) and Morse (short-range) interactions essential for capturing the crack propagation dynamics and failure modes observed in TNS’s unique trigonal structure. The selection of AIREBO-M for this study was motivated by several factors: (i) it has been extensively validated for fracture simulations in carbon-based 2D materials, demonstrating reliable reproduction of brittle fracture mechanisms and crack propagation dynamics [28,30,33]; (ii) its reactive nature explicitly captures bond breaking and formation during fracture, which is essential for studying crack propagation in TNS’s unique trigonal structure; (iii) it incorporates both short-range Morse interactions (for accurate bond dissociation) and long-range Lennard-Jones interactions (for interlayer and non-bonded interactions), providing a balanced description of the interatomic forces governing fracture; and (iv) AIREBO-M specifically addresses the high-pressure limitations of the original AIREBO potential by modifying the repulsive term, making it more suitable for studying mechanical deformation and failure [36]. Although empirical potentials have inherent limitations, AIREBO-M has been shown to predict tensile strengths for graphene (125–138 GPa [10]) that fall within the experimentally reported range (130 ± 10 GPa [1]), providing confidence in its applicability to related carbon allotropes.

The use of machine-learning interatomic potentials (MLIPs), which have recently emerged as a powerful approach to achieve DFT-level accuracy with MD-level efficiency [37]. As demonstrated by Mortazavi et al. [37], MLIPs trained on ab initio datasets can accurately reproduce mechanical properties, capture brittle fracture behavior, and enable seamless multiscale bridging from atomistics to continuum models. While we fully recognize the transformative potential of MLIPs, their application to TNS presents several challenges that fall beyond the scope of the present study: (i) training a reliable MLIP requires extensive DFT datasets encompassing diverse configurations (strained, thermally agitated, and fractured states), which are currently unavailable for TNS; (ii) the unique trigonal topology of TNS with 60° and 150° bond angles introduces complex potential energy surfaces that would require careful active learning strategies to ensure transferability; and (iii) our primary objective was to establish the first baseline mechanical property database for defective TNS, for which AIREBO-M, despite its empirical nature, provides a computationally efficient and sufficiently accurate starting point given its successful application to related carbon allotropes.

**Fig 2:**
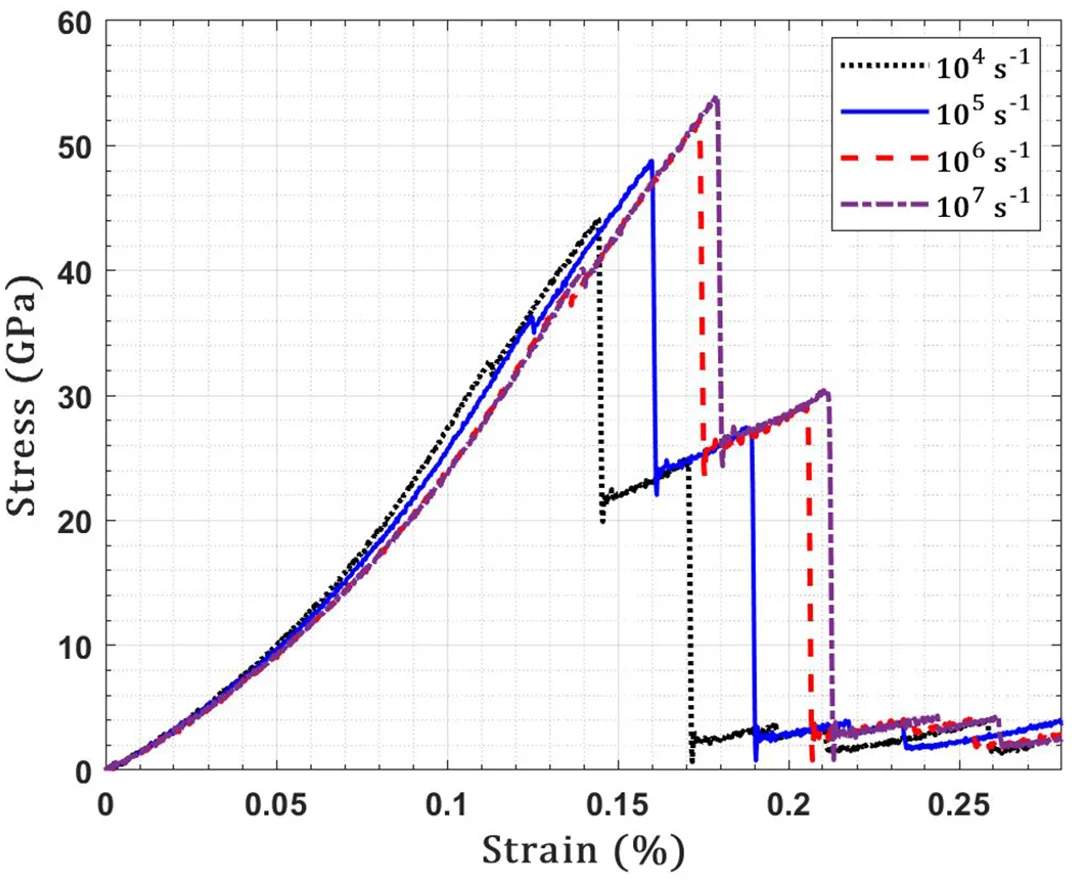
Strain rate dependence of tensile response in pre-cracked TNS nanosheet: stress-strain curves for armchair loading directions (10⁴–10⁷ s^-1^). Nanosheet: 150 Å × 150 Å with central 40 Å perpendicular crack.

This potential choice enables reliable quantification of the stress concentration effects and directional fracture mechanisms that distinguish TNS from conventional graphene behavior. Prior to mechanical testing, the system is equilibrated through a two-stage protocol: First, a 50 ps NVT ensemble simulation (constant particle number, volume, and temperature) is conducted using a Nosé-Hoover thermostat [38] to stabilize thermal conditions at 300 K. Subsequently, a 50 ps NPT ensemble simulation (constant particle number, pressure at 1 atm, and temperature) is performed to achieve proper structural relaxation. This protocol enabled comprehensive evaluation of both elastic properties and fracture mechanisms under controlled thermodynamic conditions. Single-row edge fixation (hinged boundary condition) is implemented rather than multi-row constraints, as preliminary testing revealed that multi-row fixation artificially stiffens the nanosheet and does not accurately represent realistic edge behavior [39]. This boundary condition choice directly impacts the stress distribution patterns and crack propagation mechanisms observed in our results, ensuring that the anisotropic fracture behavior reflects the intrinsic material properties rather than artificial geometric constraints. Uniaxial tensile deformation was applied along either the armchair or zigzag crystallographic direction, with transverse stress components constrained to zero to enforce a state of plane stress. Molecular dynamics simulations spanned strain rates from 10⁴ s^-1^ to 10⁷ s^-1^. Analysis identified a critical strain rate threshold of 10⁶ s^-1^, beyond which further increases exhibited negligible influence on mechanical response, indicating strain-rate insensitivity above this threshold. Fig 2 presents the corresponding engineering stress-strain curves for the pre-cracked TNS nanosheet, configured as a 150 Å × 150 Å square plate containing a centrally located 40 Å line crack oriented perpendicular to the applied uniaxial load. Data demonstrate that the maximum observed variation between stress-strain responses under the tested conditions is less than 3%. This quasi-static regime is essential for isolating the effects of crack geometry and temperature on mechanical properties, eliminating rate-dependent artifacts that could mask the fundamental anisotropic failure mechanisms central to this study.

The dimensions of the simulated nanosheet (203.23 Å × 203.23 Å) were carefully selected based on several considerations to balance computational efficiency with physical realism. First, the system size must be sufficiently large to accommodate the crack geometries studied (crack lengths up to 60 Å) while ensuring that the crack tips are adequately separated from the sheet boundaries to minimize artificial boundary effects on stress distributions. The ratio of crack length to sheet width (2a/2h) ranges from 0.15 to 0.30, which falls within the range commonly used in fracture mechanics studies of 2D materials [40,41] and ensures that the stress fields at crack tips are not significantly perturbed by the boundaries.

Second, the system must be large enough to capture the anisotropic fracture mechanisms characteristic of TNS’s unique trigonal structure. Preliminary simulations with smaller systems (100 Å × 100 Å) exhibited premature crack-boundary interactions and did not allow full development of the crack deflection phenomena observed in the Y-direction loading cases. Conversely, larger systems (300 Å × 300 Å) produced essentially identical results but required substantially more computational time (approximately 3.5 × increase), making systematic parametric studies across multiple crack angles, lengths, and temperatures computationally prohibitive. The selected size of 203.23 Å represents an optimal compromise that captures the essential physics while enabling comprehensive exploration of the parameter space.

The combination of these simulation parameters, hinged boundary conditions, strain-rate independence above 10⁶ s^-1^, and AIREBO-M potential, was validated through convergence testing and comparison with established fracture mechanics principles. This methodological framework ensures that the observed anisotropic crack deflection mechanisms and mechanical property degradation trends reflect the fundamental physics of TNS rather than simulation artifacts, providing a robust foundation for the structure-property relationships established in this work.

The mechanical properties of the trigraphene nanosheet are quantified from molecular dynamics simulations using standard post-processing procedures. The elastic modulus is determined from the initial linear portion of the stress–strain curve, typically within the first 3% strain, where the material exhibits linear elastic behavior. The modulus is calculated as the slope of this region according to the relation $E\;=\frac{d\sigma }{d\varepsilon }$, where σ and ε represent engineering stress and strain, respectively. Engineering stress is obtained from the applied force divided by the original cross-sectional area A0, with the nanosheet thickness taken as 3.4 Å (van der Waals thickness of carbon) to remain consistent with established conventions for 2D materials. The fracture strain $\left(\varepsilon_{f}\right)$ is identified as the strain corresponding to the maximum stress (ultimate tensile strength) in the stress–strain curve, marking the onset of catastrophic failure. This value is calculated as $\varepsilon_{f}\;=\;(L\;-\;L0)/L0$, where L and L0 denote the nanosheet length at failure and its initial length, respectively. The toughness is evaluated as the total strain energy absorbed per unit volume up to fracture and is obtained by numerically integrating the area under the stress–strain curve using the trapezoidal rule. Discretized stress and strain data points are used for this integration, and the toughness is approximated by the expression: $\approx \;\Sigma_{\text{i}}\;[(\sigma_{\text{i}}\;+\;\sigma_{\text{i}+\text{1}})/\text{2}]\;\times \;\left(\varepsilon_{\text{i}}^{+\text{1}}-\;\varepsilon_{\text{i}}\right)$, where $\sigma_{\text{i}}$ and $\varepsilon_{\text{i}}$ represent the stress and strain at the i-th data point, respectively. This parameter reflects the material’s energy absorption capacity prior to fracture and is expressed in units of energy per unit volume (GPa or J/m³).

Uniaxial tensile loading was applied separately along the two principal crystallographic directions of the TNS: the armchair (X) and zigzag (Y) orientations. The crack angle (θ) was defined relative to the loading direction, with θ = 0° indicating a crack aligned parallel to loading and θ = 90° indicating a perpendicular crack. Intermediate angles (e.g., 30°, 45°) were also studied to assess mixed-mode fracture behavior.

In this study, fracture toughness (${\text{K}}_{\text{c}}$) is investigated for all crack orientations relative to the applied uniaxial tensile loading direction. When the crack is oriented perpendicular to the loading direction, pure Mode I fracture behavior is observed, and the fracture toughness reduces to the Mode I critical stress intensity factor $\left({\text{K}}_{\text{c}}={\text{K}}_{\text{Ic}}\right)$. For other crack angles, mixed-mode conditions (combining Modes I and II) may arise, and ${\text{K}}_{\text{c}}$ represents the effective fracture toughness under combined loading. The critical stress intensity factor (${\text{K}}_{\text{c}}$) at the onset of fracture (corresponding to the ultimate tensile strength) is calculated using the following expression for a central crack in a finite-width nanosheet [40,41]:


$$\begin{array}{c} {\text{K}}_{\text{c}}=\sigma_{f}\sqrt{\text{2}htan(\frac{\pi a}{\text{2}h}}) \end{array}$$ (1)

where $\sigma_{f}$ is the maximum tensile stress, and 2h is the width of the nanosheet, and 2a is the initial crack length.

In addition to the critical stress intensity factor, we compute the critical energy release rate (${\text{G}}_{\text{c}}$), which represents the energy dissipated per unit area during crack propagation. For a material under plane stress conditions, ${\text{G}}_{\text{c}}$ is related to ${\text{K}}_{\text{c}}$ through the elastic modulus E by [42]:


$$\begin{array}{c} {\text{G}}_{\text{c}}=\frac{{\text{K}}_{\text{c}}^{\text{2}}}{\text{E}} \end{array}$$ (2)

where E is the orientation-dependent elastic modulus of the pristine material. This relationship assumes linear elastic behavior up to fracture, which is consistent with the brittle fracture mechanisms observed in TNS under X-direction loading. For mixed-mode conditions (e.g., Y-direction loading with crack deflection), this expression provides an effective energy release rate that accounts for the combined fracture modes.

To address the inherent statistical uncertainties arising from thermal fluctuations in molecular dynamics simulations, each simulation condition was repeated five times with different initial velocity distributions generated from independent random seeds corresponding to the target temperature (Maxwell-Boltzmann distribution). For each set of parameters (crack angle, crack length, temperature, and loading direction), the mean value and standard deviation were computed from these five independent trajectories. The error bars presented in all figures.

## 3. Result and discussion

**Fig 3:**
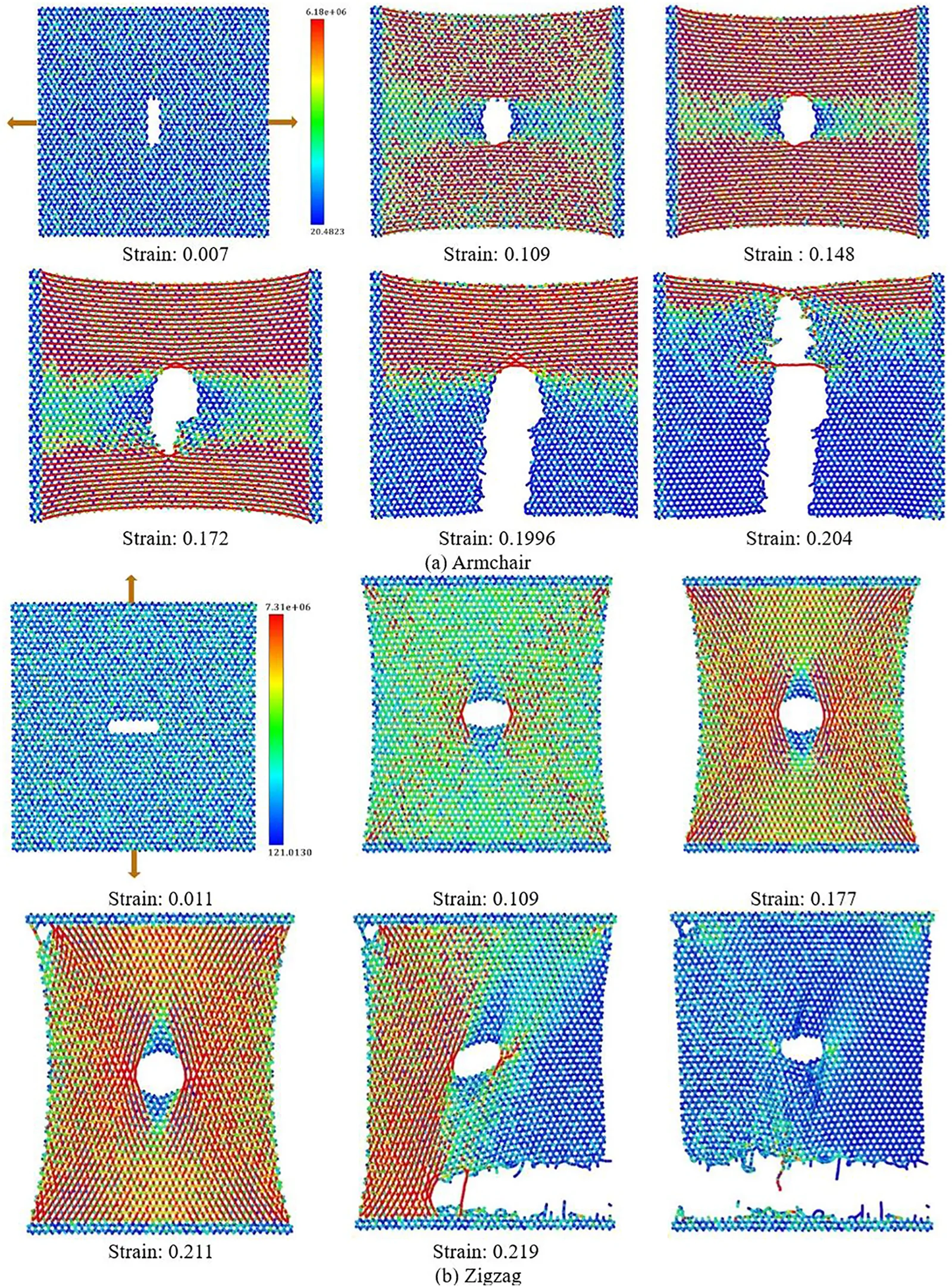
Atomistic evolution of fracture in TNS under uniaxial loading with a central crack-oriented perpendicular to the loading direction. (a) Armchair, (b) Zigzag.

Fig 3 illustrates the fracture evolution process in a TNS containing a central crack-oriented perpendicular to the loading direction, analyzed under uniaxial tensile loading in both X and Y directions. Various stages of deformation are identified by increasing strain levels, ranging from initial elastic stretching to final fracture. The distinct behavior in each direction is a clear indication of anisotropic mechanical response and fracture patterns. In the X direction (Armchair loading), fracture develops symmetrically along the crack line, indicating that stress is highly concentrated at the crack tips due to the tensile load being aligned with the crack propagation path. As strain increases, localized deformation accumulates at the crack tips, and bond breaking initiates there, leading to a clean crack extension through the sheet. The smooth vertical separation observed at strain levels around 0.204% reflects a brittle fracture mechanism governed by mode I (opening mode) stress intensity, where the crack opens due to direct tensile stress normal to the crack surface. This pattern confirms a high stress concentration along the crack plane, resulting in a dominant crack growth trajectory aligned with the loading direction. In contrast, under Y direction loading (Zigzag direction), although the crack is initially perpendicular to the applied stress, the fracture path diverges from the crack line and propagates toward the sheet boundaries. This behavior, clearly visible at strain levels above 0.21, suggests that the stress distribution is more diffused, and the crack tip shielding effect is more significant in this orientation. Instead of continuing along the initial crack path, stress concentrations shift toward weaker regions near the edges, leading to crack branching or deflection toward the lateral boundaries of the nanosheet. This indicates the stress intensity factor at the crack tip is insufficient to drive a straight-line propagation, and instead, boundary-induced stress amplification initiates fracture at off-center regions. The observed differences in crack propagation behavior between the two directions underscore the role of atomic orientation in stress transfer and failure mechanics. The X direction, with its aligned carbon-carbon bonds, provides a more direct stress transmission path to the crack tip, promoting brittle and localized failure. Meanwhile, the Y direction involves more angular atomic paths that dissipate stress more broadly, resulting in delayed and deflected fracture.

The observed anisotropic fracture behavior in TNS is fundamentally rooted in its unique trigonal atomic structure and the resulting directional variations in bond strength and stress distribution mechanisms. Unlike graphene’s uniform sp² hybridization with 120° bond angles, TNS exhibits significant angular distortions with characteristic angles of 60° (C1-C3-C4) and 150° (C4-C6-C5), creating inherent structural asymmetry. Under X-direction loading, the stress transmission pathway aligns more favorably with the stronger inter-trigon bonds (1.349 Å), enabling efficient load transfer directly to the crack tip. This alignment results in highly localized stress concentrations that exceed the local bond strength threshold, leading to catastrophic bond rupture and symmetric crack propagation. Conversely, Y-direction loading forces stress to traverse through the weaker intra-trigon bonds (1.419 Å) and the distorted angular network, causing stress to redistribute over a broader area. The tortuous stress path through the zigzag configuration dissipates energy more effectively, reducing peak stress concentrations at the crack tip and enabling alternative failure pathways through crack deflection toward structural boundaries where stress redistribution creates new failure nucleation sites.

It is important to acknowledge that edge effects and stress concentrations near fixed boundaries are inherent limitations of finite-sized molecular dynamics simulations and cannot be entirely eliminated. In the present simulations, fixed boundary conditions are applied at the specimen edges, introducing artificial constraints that influence the local stress distribution, particularly in regions adjacent to the boundaries. The degree of edge effect is found to vary with loading direction, as evidenced by the results. Under Y-direction loading (Fig 3(b)), the crack propagation path is observed to exhibit more pronounced deviations and localized stress concentrations near the constrained atomic rows compared to the X-direction loading cases. This directional dependence reflects the anisotropic influence of the boundaries on crack evolution and stress redistribution. These stress concentrations are naturally developed during the loading process and are inherently captured in the simulation outputs. Although such effects arise from the finite domain size and do not represent intrinsic material properties, they do not substantially alter the dominant fracture mechanisms under investigation, including crack deflection, branching, and interactions with twin boundaries. Thus, while quantitative stress values near boundaries should be interpreted with appropriate caution, the primary fracture behaviors remain representative of the material’s intrinsic response.

A closer examination of the fracture evolution under Y-direction loading reveals a noteworthy feature: at strain = 0.211, initial damage appears near one side of the nanosheet, yet catastrophic failure ultimately occurs on the opposite side at strain = 0.219. This behavior, while perhaps unexpected, is a direct consequence of the diffuse stress distribution characteristic of zigzag loading in TNT’s trigonal structure. Under Y-direction loading, the stress transmission pathway must navigate through the distorted angular network of 60° and 150° bonds, creating a more diffuse stress field compared to the highly localized crack-tip concentration observed under X-direction loading. In this diffuse field, multiple locations within the nanosheet experience comparable stress levels, and damage can initiate at several sites sequentially. The initial damage observed at strain = 0.211 represents localized bond breakage, likely at a region of higher local stress concentration near the boundary, but this damage does not immediately propagate catastrophically because the surrounding atomic configuration and stress field do not yet favor continuous crack growth. Instead, stress redistributes, and the diffuse field gradually intensifies elsewhere. Between strain = 0.211 and 0.219, stress continues to build, and the critical threshold for catastrophic failure is eventually reached at the opposite side of the sheet, where a combination of the crack-tip stress field and boundary-induced stress concentration creates the most energetically favorable path for rapid crack propagation. The earlier damage site remains as a non-propagating microcrack or void, consistent with the concept of multiple site damage observed in heterogeneous materials. This sequence of events, multiple damage initiation sites followed by failure at the most critical location, is a hallmark of materials with diffuse stress distributions and is amplified in TNS by its unique trigonal topology. It is important to note that this behavior is not an artifact of the hinged boundary conditions, as similar diffuse damage accumulation has been observed in other 2D materials with complex bond networks [43].

To establish confidence in our simulation methodology for defective TNS, we first evaluate the mechanical properties of pristine (crack-free) trigraphene nanosheets and compare them with recent molecular dynamics studies available in the literature. Table 1 summarizes the elastic modulus and ultimate tensile strength for pristine TNS under uniaxial tension along both armchair (X) and zigzag (Y) directions at 300 K, obtained using the same AIREBO-M potential and simulation protocol described in Section 2. These values are compared with those reported by Li and Su [44] for TNS of comparable dimensions.

**Table 1:** Mechanical properties of pristine TNS at 300 K: comparison with Li and Su [44].

Property	This Study (MD)	Li and Su [44]	Deviation (%)
Elastic Modulus (X)	215.3 GPa	191.6 GPa (at 150 Å)	+12.4%
Elastic Modulus (Y)	209.7 GPa	186.1 GPa (at 150 Å)	+12.7%
Ultimate Strength (X)	82.4 GPa	87.5 GPa (at 150 Å)	−5.8%
Ultimate Strength (Y)	94.7 GPa	102.0 GPa (at 150 Å)	−7.2%

As shown in Table 1, our predictions for ultimate tensile strength are in reasonable agreement with those reported by Li and Su [44], with deviations of approximately 6–7%. The slightly lower strength values in our study may be attributed to the larger nanosheet dimensions used in our work (203.23 Å vs. 150 Å), as Li and Su demonstrated a clear decreasing trend in strength with increasing side length due to the higher probability of encountering weak spots in larger samples. The elastic modulus values obtained in this study are approximately 12–13% higher than those reported by Li and Su [44]. This difference likely stems from variations in the specific implementation of the AIREBO-M potential, equilibration protocols, or strain rates employed in the two studies. Nonetheless, both studies confirm the same anisotropic trends: the Y (zigzag) direction exhibits higher ultimate strength than the X (armchair) direction, while the X direction shows slightly higher elastic modulus. This consistency in directional dependence validates the fundamental physical behavior captured by our simulations.

The elastic modulus values obtained for pristine TNS (215.3 GPa in X-direction, 209.7 GPa in Y-direction) are substantially lower than those of graphene (~1 TPa [1]) but comparable to other porous carbon allotropes. For example, penta-graphene has been reported to have an elastic modulus of approximately 264 GPa [9], while phagraphene exhibits values around 320 GPa [6]. The lower modulus of TNS reflects its lower bond density per unit area due to the presence of larger rings in its structure. The anisotropy ratio (${\text{E}}_{X}/{\text{E}}_{Y}=\text{1}.\text{03}$) in TNS is relatively small compared to other 2D materials. Graphene exhibits nearly isotropic elastic behavior (${\text{E}}_{Armchair}/{\text{E}}_{Zigzag}$≈ 1.0) due to its perfect hexagonal symmetry [1]. In contrast, penta-graphene shows stronger anisotropy with reported ratios of 1.1–1.2 depending on the specific pentagonal arrangement [9]. The moderate anisotropy in TNS arises from the asymmetric distribution of 60° and 150° bond angles, which creates different effective stiffness along the two crystallographic directions.

To assess whether our conclusions are sensitive to absolute system size, we performed additional simulations for selected crack configurations (crack length = 40 Å, angle = 90°) using a smaller system (150 Å × 150 Å) and a larger system (300 Å × 300 Å). The ultimate strength values for the 203.23 Å and 300 Å systems agree within 2.1% for the X-direction and 1.8% for the Y-direction, while the 150 Å system shows slightly lower values (8–9% reduction) due to premature crack-boundary interactions. The crack deflection angle in Y-direction loading differs by less than 3° between the 203.23 Å and 300 Å systems, confirming that the observed fracture mechanisms are not artifacts of finite size. Based on these convergence tests, we conclude that the 203.23 Å system size is sufficient to capture size-independent fracture behavior for the crack lengths studied.

To assess whether our conclusions are sensitive to absolute system size, we performed additional simulations for selected crack configurations (crack length = 40 Å, angle = 90°) using a smaller system (150 Å × 150 Å) and a larger system (300 Å × 300 Å). As shown in Table 2, the ultimate strength values for the 203.23 Å and 300 Å systems agree within 2.1% for the X-direction and 1.8% for the Y-direction, while the 150 Å system shows slightly lower values (4–5% reduction) due to premature crack-boundary interactions. The crack deflection angle in Y-direction loading differs by less than 3° between the 203.23 Å and 300 Å systems, confirming that the observed fracture mechanisms are not artifacts of finite size. Based on these convergence tests, we conclude that the 203.23 Å system size is sufficient to capture size-independent fracture behavior for the crack lengths studied.

**Table 2:** Critical energy release rate $G_{c}$ (J/m²) of TNS under various conditions.

Condition	Value	Armchair (X)	Zigzag (Y)
Crack angle (crack length = 40 Å, T = 300 K)	0°	148.0	201.1
	30°	132.8	190.0
	45°	117.3	160.8
	60°	106.9	148.7
	90°	94.2	120.9
Crack length (crack angle = 90°, T = 300 K)	30 Å	159.8	205.6
	40 Å	94.2	120.9
	45 Å	58.9	85.7
	50 Å	52.4	72.1
	60 Å	45.2	64.7
Temperature (crack length = 40 Å, crack angle = 90°)	200 K	199.4	258.4
	300 K	94.2	120.9
	400 K	76.4	105.1
	500 K	59.2	89.6
	600 K	49.2	69.2
	700 K	39.7	49.9
	800 K	35.6	43.9
	900 K	31.6	38.1
	1000 K	27.8	32.6

**Fig 4:**
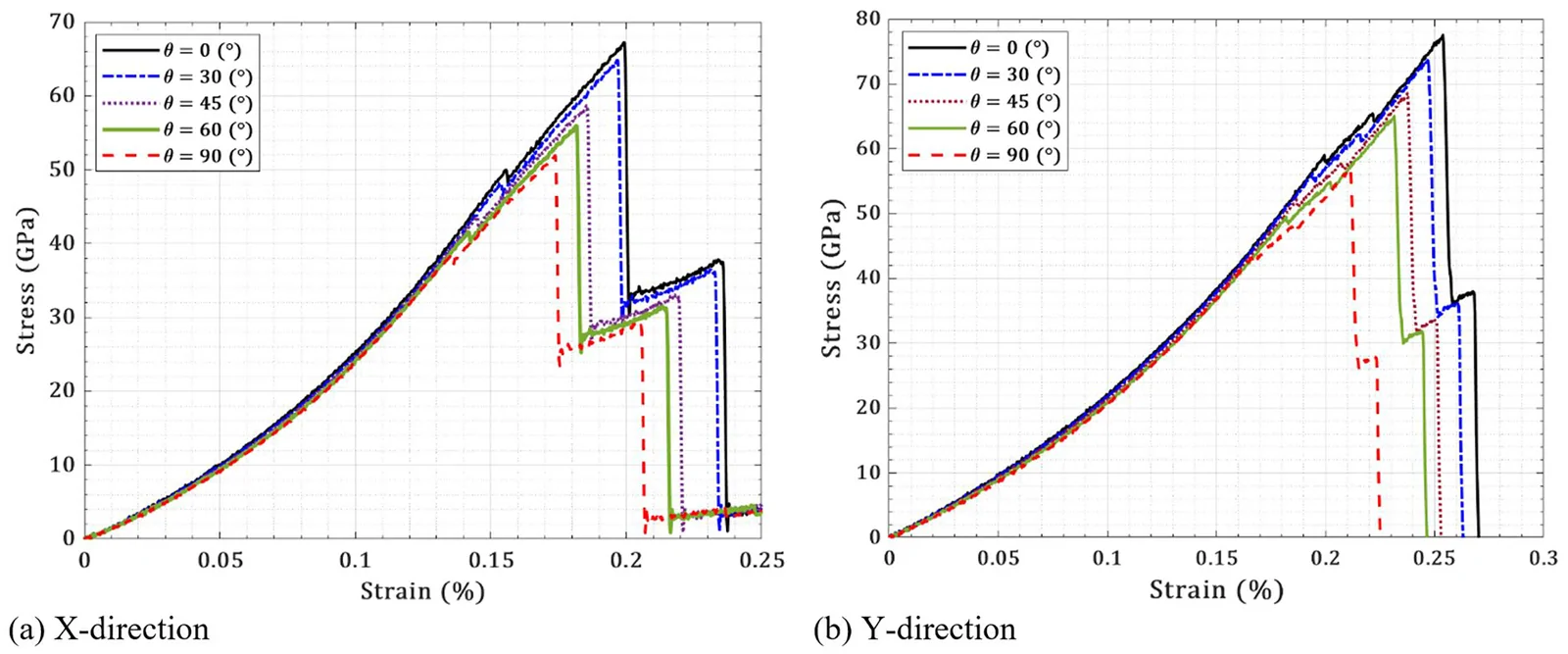
Stress-strain curve of TNS as a function of crack angle. (a) X-direction, (b) Y-direction.

The stress-strain behavior of square TNS with a side length of 203.23 Å, containing a central crack of 40 Å in length, is investigated under uniaxial tensile loading along both the armchair (X) and zigzag (Y) directions at 300 K. The study examines the influence of crack angle from 0° to 90° with respect to the loading axis, as illustrated in Fig 4. The data demonstrate a systematic degradation in mechanical properties, particularly the elastic modulus and ultimate tensile strength, as the crack angle increases, a trend observed in both the X and Y loading directions. This reduction is attributed to the altered stress distribution and localized strain concentration near the crack tip, which becomes more pronounced at higher crack angles. Furthermore, the stress-strain curves exhibit pronounced anisotropic characteristics between the X and Y directions, reflecting distinct deformation mechanisms. Specifically, the Y direction displays a higher resistance to crack propagation compared to the X direction, leading to variations in failure thresholds.

**Fig 5:**
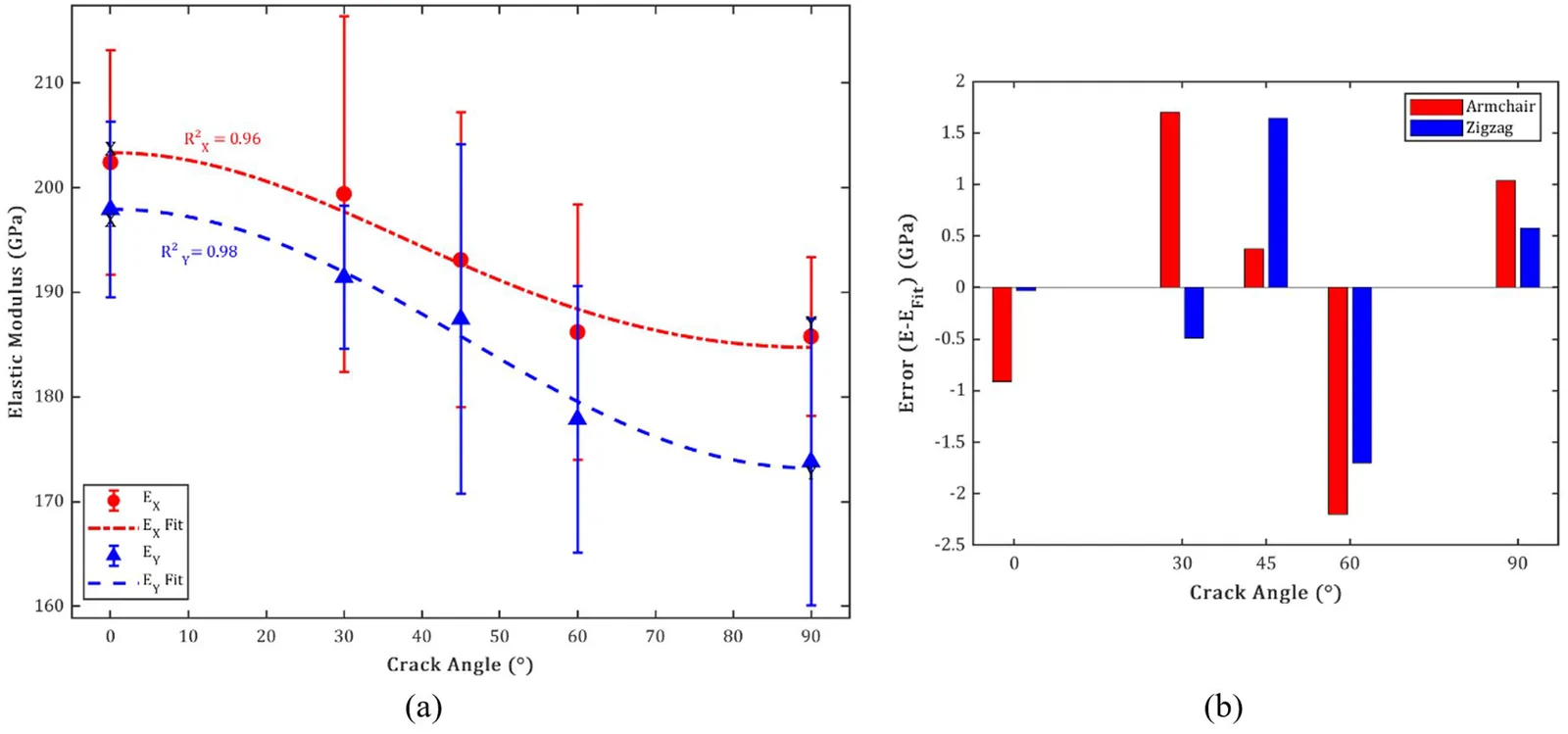
(a) Variation of elastic modulus with crack angle (b) elastic modulus fitting error for TNS under uniaxial loading at 300 K.

Fig 5 illustrates the variation of elastic modulus of a TNS with a central crack of length 40 Å, embedded within a sheet of side length 203.23 Å, subjected to uniaxial loading at 300 K. The elastic modulus values are computed based on the 5% offset criterion from the initial linear portion of the stress-strain curve. The data is presented for both Armchair (X direction) and Zigzag (Y direction) loading scenarios across varying crack propagation angles: 0°, 30°, 45°, 60°, and 90°. The finding reveal that the elastic modulus decreases with increasing crack angle in both directions, indicating a progressive degradation of the material’s ability to resist elastic deformation as the crack becomes more inclined. This degradation is more pronounced between 0° and 60°, and tends to saturate near 90°. For the X direction, the elastic modulus decreases from 202.43 GPa at 0° to 185.78 GPa at 90°, indicating an 8.23% reduction over the full angular range. For the Y direction, it decreases from 197.92 GPa at 0° to 173.79 GPa at 90°, amounting to a 12.19% reduction. These values indicate that the elastic modulus in the Y direction is more sensitive to crack angle than in the X direction, suggesting that Zigzag-oriented structures are mechanically more susceptible to crack-induced degradation. Cracks aligned with the loading direction (0°) disrupt fewer load-bearing bonds, resulting in higher stiffness, while angled cracks (especially at 45°–60°) intersect more of the critical stress paths, weakening the structure more significantly. The findings also underscore the anisotropic mechanical behavior of trigraphene. The X direction consistently exhibits higher elastic modulus than the Y direction across all crack angles. This anisotropy likely originates from the directional dependency of the bond structure and atomic alignment in the 2D lattice, where the Armchair direction may support more efficient load transfer due to the alignment of strong σ bonds. The variation of elastic modulus with crack orientation angle θ in anisotropic or cracked materials can be accurately represented by the trigonometric expression: $\text{E}(\theta )={\text{E}}_{\text{0}}+{\text{E}}_{\text{1}}\text{cos}(\text{2}\theta )+{\text{E}}_{\text{2}}\text{cos}(\text{4}\theta )$. This functional form effectively captures the periodic and symmetric nature of the elastic response as a function of crack direction, particularly in materials exhibiting orthotropic or higher-order anisotropy. For instance, in the armchair direction, the elastic modulus is fitted as ${\text{E}}_{\text{X}}=\text{193}.\text{4}+\text{9}.\text{3cos}(\text{2}\theta )+\text{0}.\text{7cos}(\text{4}\theta )$, yielding a coefficient of determination ${\text{R}}^{\text{2}}=\text{0}.\text{96}$, whereas in the zigzag direction, the corresponding fit is ${\text{E}}_{\text{Y}}=\text{185}.\text{7}+\text{12}.\text{4cos}(\text{2}\theta )-\text{0}.\text{1cos}(\text{4}\theta )$ with ${\text{R}}^{\text{2}}=\text{0}.\text{98}$, indicating excellent agreement with the numerical data. This modeling approach is grounded in classical elasticity theory and homogenization principles for cracked or directionally heterogeneous media. The angular dependence of stiffness or compliance tensors in such systems can be systematically expanded as a Fourier series involving even multiples of the crack orientation angle, consistent with the underlying material symmetries [45].

The systematic degradation of mechanical properties with increasing crack angle can be attributed to the progressive misalignment between the crack geometry and the principal stress trajectories within the TNS lattice. At θ = 0°, the crack lies parallel to the loading direction, minimizing stress concentration by preserving the maximum number of load-bearing bonds perpendicular to the crack plane. As the crack angle increases toward 90°, the crack increasingly intersects the primary stress-carrying bonds, creating a dual effect: (1) reduction in the effective cross-sectional area available for load transfer, and (2) intensification of stress concentrations at the crack tips due to geometric stress multiplication factors. The trigonometric relationship $\text{E}(\theta )={\text{E}}_{\text{0}}+{\text{E}}_{\text{1}}\text{cos}(\text{2}\theta )+{\text{E}}_{\text{2}}\text{cos}(\text{4}\theta )$ observed in the elastic modulus reflects the underlying crystallographic symmetry, where the cos(2θ) term captures the primary anisotropy between armchair and zigzag directions, while the cos(4θ) term accounts for higher-order effects related to the trigonal symmetry of the TNS structure. The greater sensitivity in the Y-direction (larger ${\text{E}}_{\text{1}}$ coefficient) indicates that zigzag-oriented loading is more susceptible to crack-induced stress field perturbations due to the inherent structural compliance of the trigonal network.

**Fig 6:**
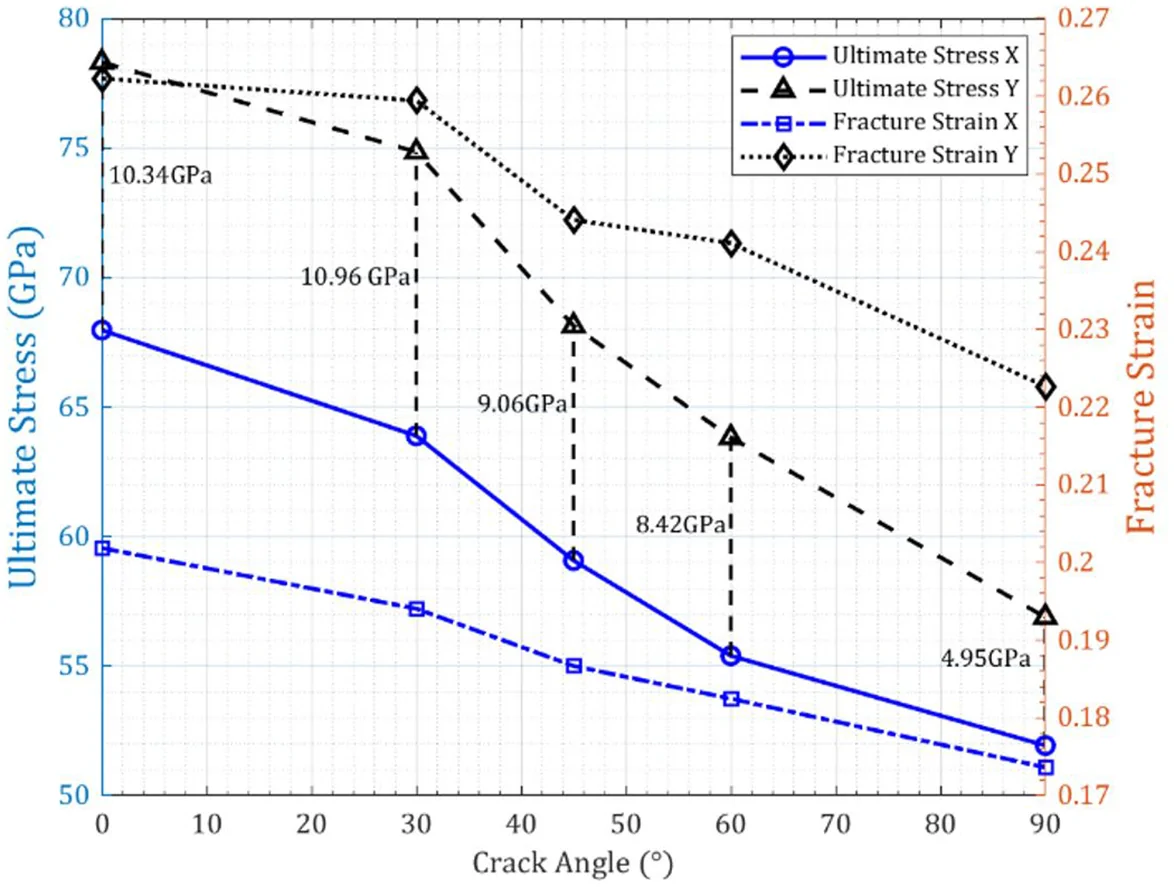
Variation of ultimate stress and fracture strain of TNS with crack angle at 300K.

Fig 6 illustrates the variation of ultimate stress and fracture strain of a TNS with a centrally located crack (length = 40 Å) under uniaxial tension along both armchair (X) and zigzag (Y) directions at 300 K, as the crack angle varies from 0° to 90°. In the armchair direction, the ultimate stress decreases from 67.97 GPa to 51.93 GPa, a reduction of approximately 23.6%, while the fracture strain drops by about 14%. In the zigzag direction, the ultimate stress declines from 78.31 GPa to 56.89 GPa (a 27.4% decrease), and the fracture strain reduces by around 15.1%. Across all angles, the zigzag direction exhibits higher ultimate stress and fracture strain, indicating superior strength and ductility due to anisotropic lattice characteristics. The analysis confirms that increasing the crack angle weakens the structure by misaligning the crack path with the principal load direction, leading to reduced mechanical performance.

The observed behavior, where the elastic modulus in the X direction is higher than in the Y direction, while the ultimate stress is greater in the Y direction. The higher elastic modulus in the X direction suggests that the atomic arrangement along the armchair orientation provides greater initial stiffness and resistance to small deformations, likely due to tighter bond packing and more linear load paths. However, the higher ultimate stress in the Y direction indicates that, despite being initially less stiff, the zigzag orientation can endure greater loads before failure. This is likely because the zigzag path offers more effective energy dissipation and resistance to crack propagation under large deformations, possibly due to more flexible or reorientable atomic bonding patterns.

**Fig 7:**
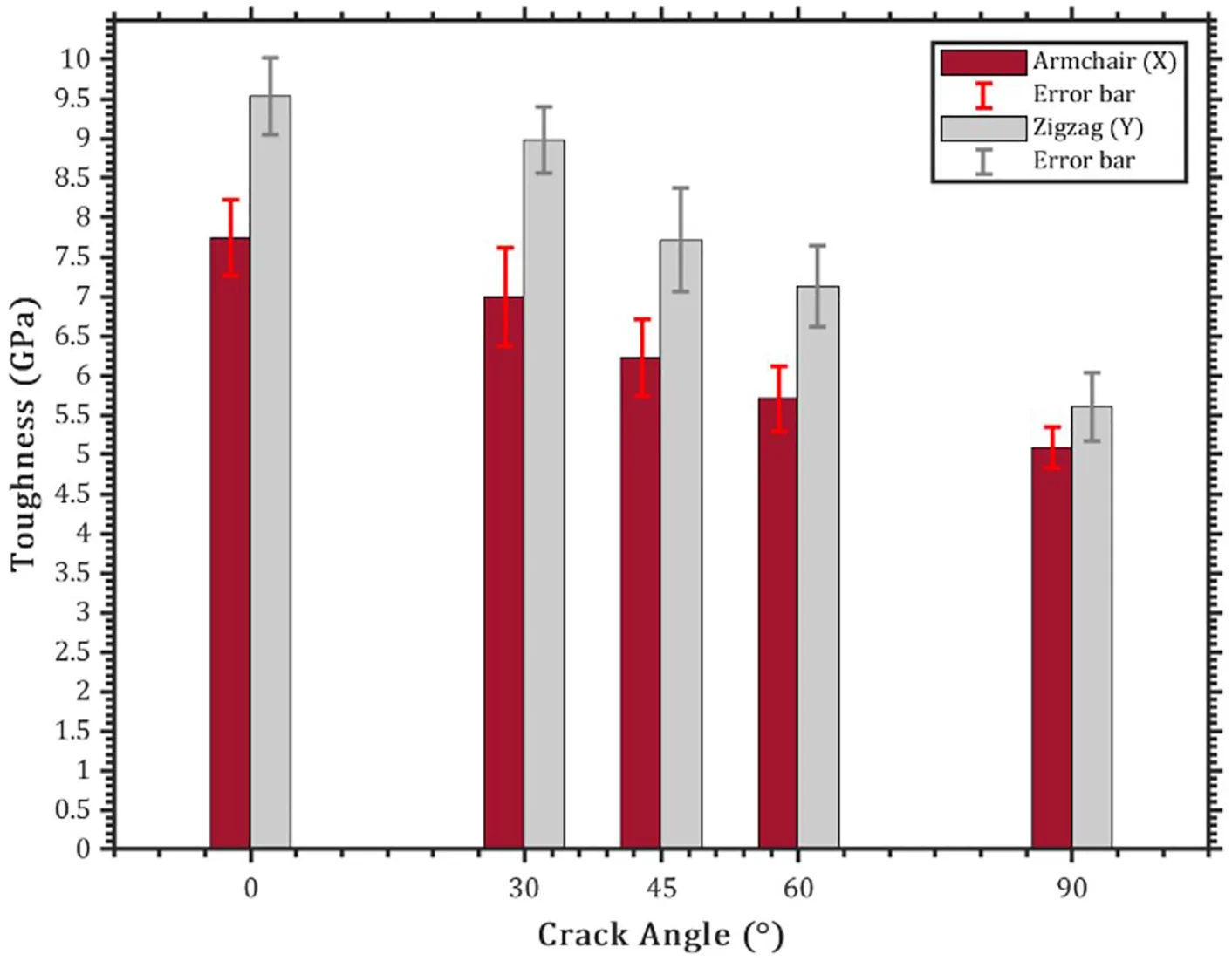
Variation of toughness of TNS with crack angle at 300K.

Fig 7 illustrates the variation in toughness (measured in GPa as energy per unit volume) of a TNS with a central crack of length 40 Å, subjected to different crack orientations (0°–90°) at 300 K. In the X direction, toughness decreases from 7.74 to 5.09 GPa, a ~ 34.3% drop, while in the Y direction, it falls from 9.53 to 5.60 GPa, a ~ 41.3% reduction. Although the zigzag direction consistently exhibits higher toughness, suggesting greater energy absorption capacity before failure, it also demonstrates more sensitivity to crack orientation. These data emphasize the anisotropic nature of TNS.

**Fig 8:**
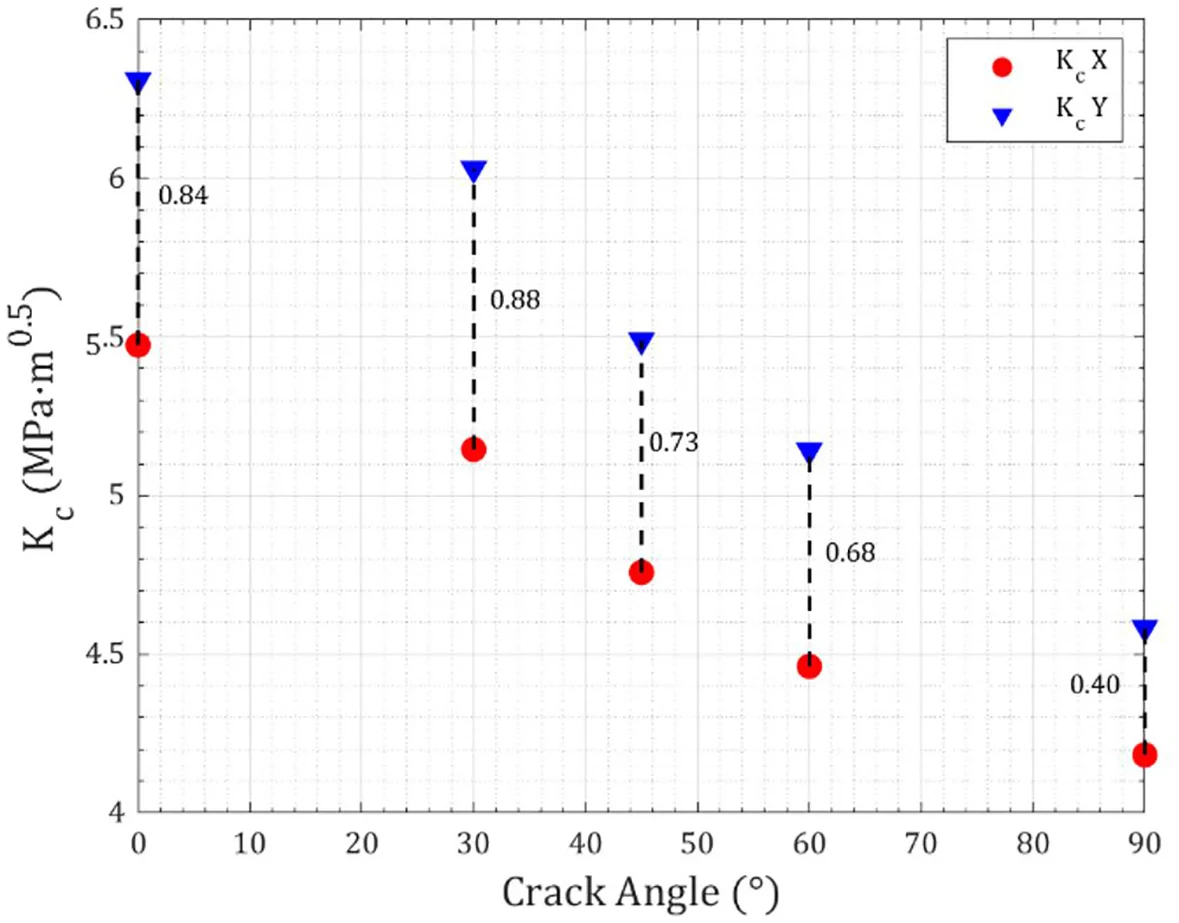
Variation of stress intensity factor of TNS with crack angle at 300K.

The variation of the stress intensity factor (${\text{K}}_{\text{c}},\text{ MPa}\cdot \sqrt{\text{m}}$) of TNS with a crack located at the center of the nanosheet (side length 203.23 Å) and crack length of 40 Å, under varying crack angles (at T = 300 K), is shown in Fig 8. The data reveals that, both the X and Y directions exhibit a decrease in ${\text{K}}_{\text{Ic}}$ as the crack angle increases from 0° to 90°. The X-direction experiences a reduction of approximately 23.7%, while the Y-direction sees a larger decrease of about 27.3%. The Armchair direction, aligned with the graphene’s intrinsic atomic bonding, typically provides higher mechanical strength, as the bonds along this direction are stronger, resulting in a higher ${\text{K}}_{\text{Ic}}$ at 0°. As the crack angle increases, the crack propagation becomes less favorable in the X direction due to the mismatch between the crack path and the crystal structure, leading to a more significant reduction in fracture toughness. Conversely, the Y direction, where the atomic bonds are more susceptible to crack propagation, experiences a greater initial fracture toughness but loses strength more rapidly as the crack angle increases. The Y direction’s greater reduction in ${\text{K}}_{\text{Ic}}$ suggests that cracks align more easily with the atomic planes, leading to easier propagation and thus a more significant reduction in fracture resistance.

**Fig 9:**
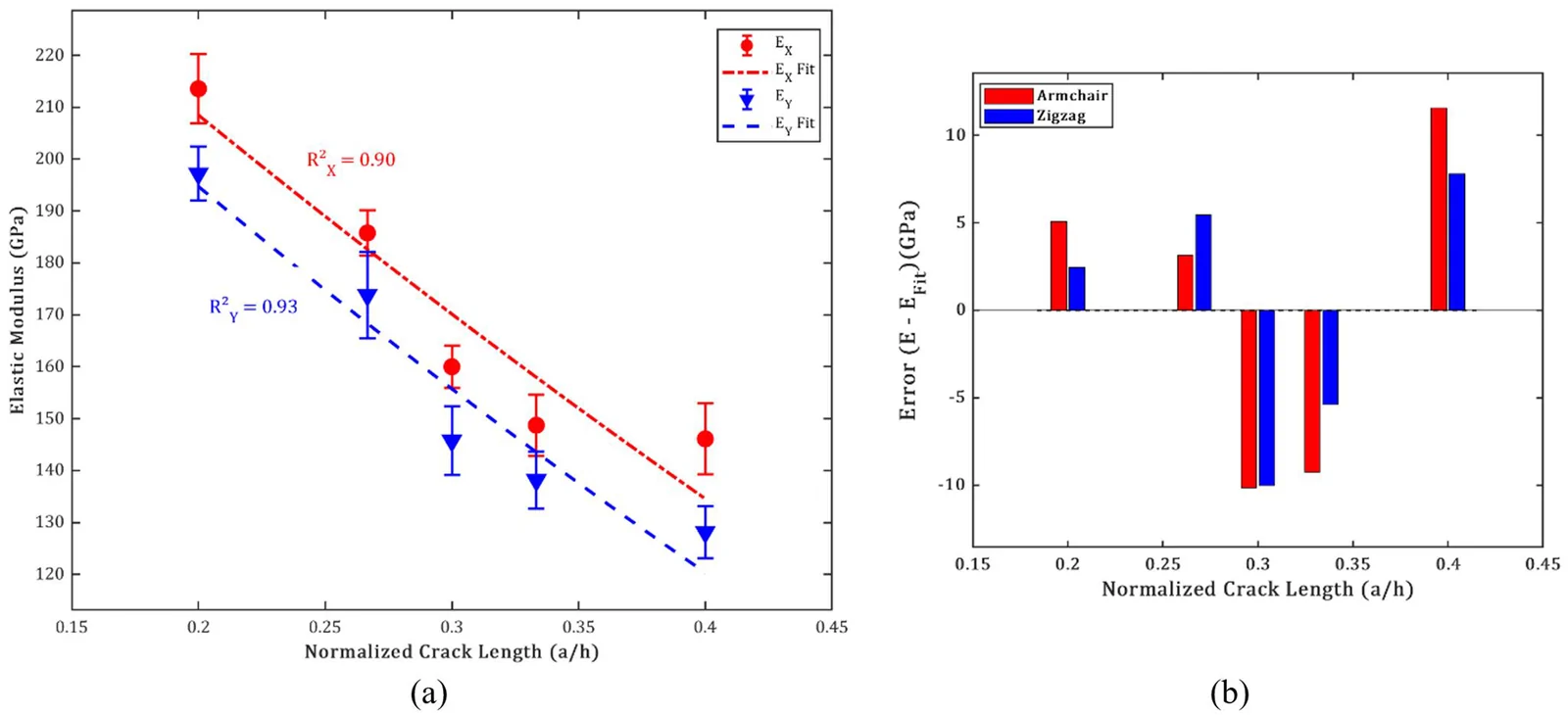
(a) Variation of elastic modulus with crack length (b) elastic modulus fitting error for TNS under uniaxial loading at 300 K.

The variation of the elastic modulus of a TNS with a crack located around the middle of the nanosheet, with a side length of 203.23 Å and a crack angle of 90°, under varying crack lengths (at T = 300 K), is shown in Fig 9. The study reveals that as the crack length increases, the elastic modulus decreases in both the X and Y directions, reflecting the expected reduction in mechanical stiffness due to crack propagation. In the X direction, the elastic modulus decreases from 213.57 GPa at a crack length of 30 Å to 146.10 GPa at 60 Å. In the Y direction, a similar trend is observed, with the modulus reducing from 197.20 GPa at 30 Å to 128.12 GPa at 60 Å. The reduction in modulus is more pronounced in the X direction, which suggests that the crack influences the mechanical properties of the nanosheet more significantly along the armchair direction than the zigzag direction. The generalized power-law expression $\text{E}={\text{E}}_{\text{0}}{\left(\text{1}-\frac{\text{a}}{h}\right)}^{n}$ is widely employed in fracture mechanics and materials science to characterize the degradation of elastic modulus with increasing crack length. This form is consistent with nonlinear failure criteria observed in fractured and damaged materials, such as rocks under triaxial stress conditions, where power-law relationships effectively describe the evolution of shear strength [46]. In the present analysis, this model was applied to capture the variation of elastic modulus with normalized crack length a/h. In the armchair direction, the fitted relation is: $\text{E}=\text{292}.\text{8}{\left(\text{1}-\frac{\text{a}}{h}\right)}^{\text{1}.\text{52}}$ with a coefficient of determination ${\text{R}}^{\text{2}}=\text{0}.\text{9}$, while in the zigzag direction, the fit yields: $\text{E}=\text{282}.\text{9}{\left(\text{1}-\frac{\text{a}}{h}\right)}^{\text{1}.\text{67}}$ with ${\text{R}}^{\text{2}}=\text{0}.\text{93}$, both indicating strong agreement between the model and the computed data. These results affirm the suitability of the power-law framework for describing the stiffness reduction due to crack growth in anisotropic nanoscale systems.

The substantial degradation of mechanical properties with increasing crack length follows classical fracture mechanics principles but is amplified by the nanoscale effects unique to TNS. The power-law relationship reflects the progressive reduction in load-bearing capacity as the crack consumes an increasing fraction of the structural cross-section. However, the nanoscale dimensions of TNS introduce additional mechanisms beyond simple area reduction. As crack length increases, the stress concentration factor at the crack tip intensifies according to the relationship $K=\sigma \sqrt{\left(\pi a\right)}$, where longer cracks create sharper stress gradients that exceed the local bond strength over larger regions. The trigonal structure of TNS exacerbates this effect because the distorted bond network creates preferential failure pathways that propagate more readily under high stress gradients. Furthermore, the discrete atomic structure means that crack tip stress fields interact with individual atomic bonds rather than being averaged over a continuum, leading to more brittle failure behavior. The similar degradation rates in both X and Y directions (n ≈ 1.5–1.7) suggest that while the absolute strength differs between directions, the fundamental crack propagation mechanics are governed by similar bond-breaking processes once the critical stress threshold is exceeded.

**Fig 10:**
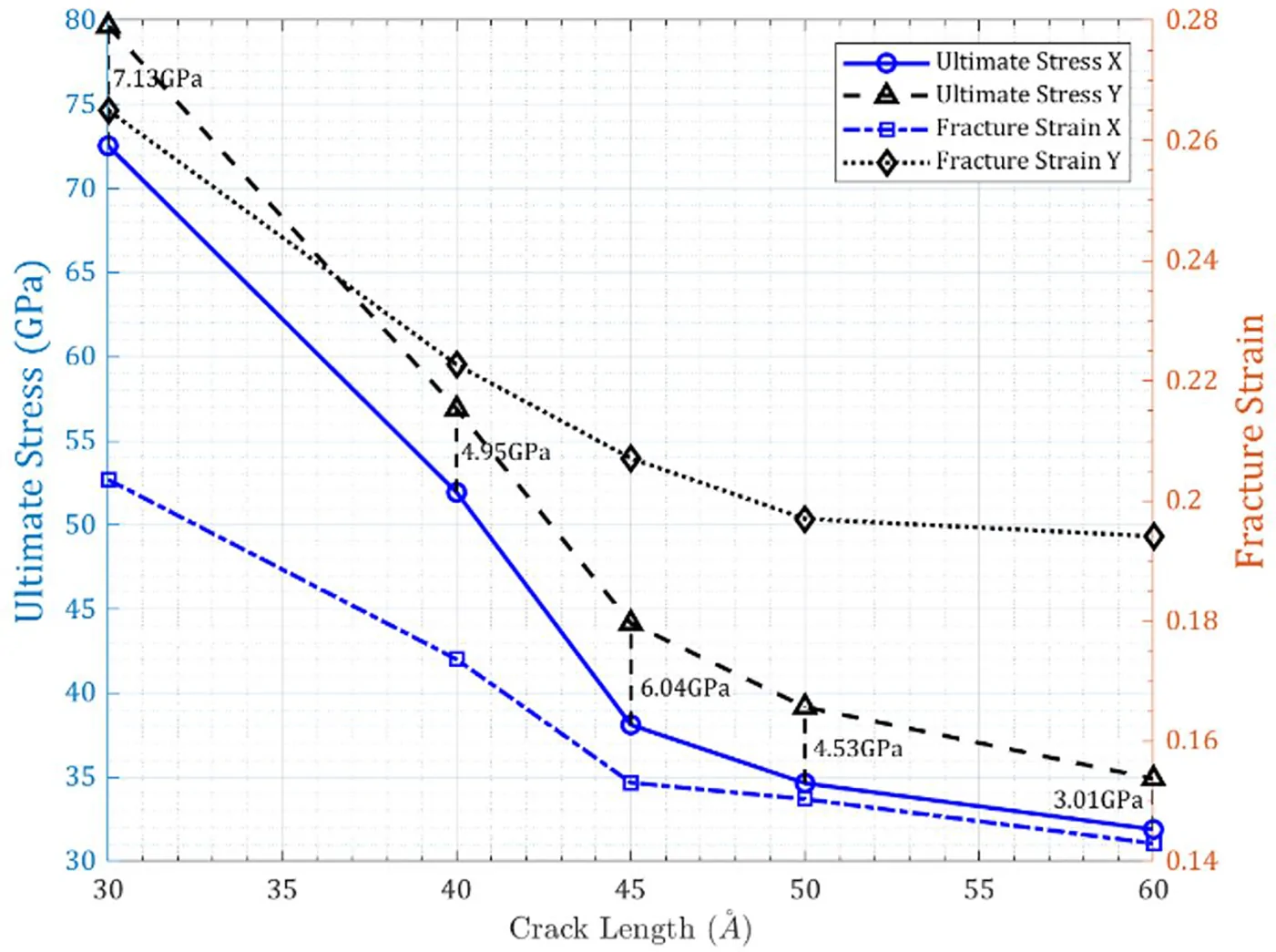
Variation of ultimate stress and fracture strain of TNS with crack length at 300K.

Fig 10 illustrates the variation of ultimate stress and fracture strain of a TNS with a central crack (crack angle 90°) under different crack lengths at a temperature of 300 K. As the crack length increases from 30 Å to 60 Å, a notable decline in both ultimate stress and fracture strain is observed in both X and Y directions. Specifically, the ultimate stress in the X direction drops from 72.54 GPa to 31.89 GPa, a reduction of approximately 56%, while in the Y direction, it decreases from 79.67 GPa to 34.91 GPa, about 56% as well. Similarly, the fracture strain in the X direction declines from 0.204 to 0.143 (≈30% decrease), whereas in the Y direction it decreases from 0.265 to 0.194 (≈27%). The consistent percentage reduction in both mechanical properties across directions signifies a substantial degradation in the nanosheet’s resistance to failure as the crack grows. These reductions are attributed to the increased stress concentration and reduced load-bearing area due to crack propagation, which weakens the structural integrity of the nanosheet. Notably, the Y direction consistently exhibits higher values of both ultimate stress and fracture strain than the X direction.

**Fig 11:**
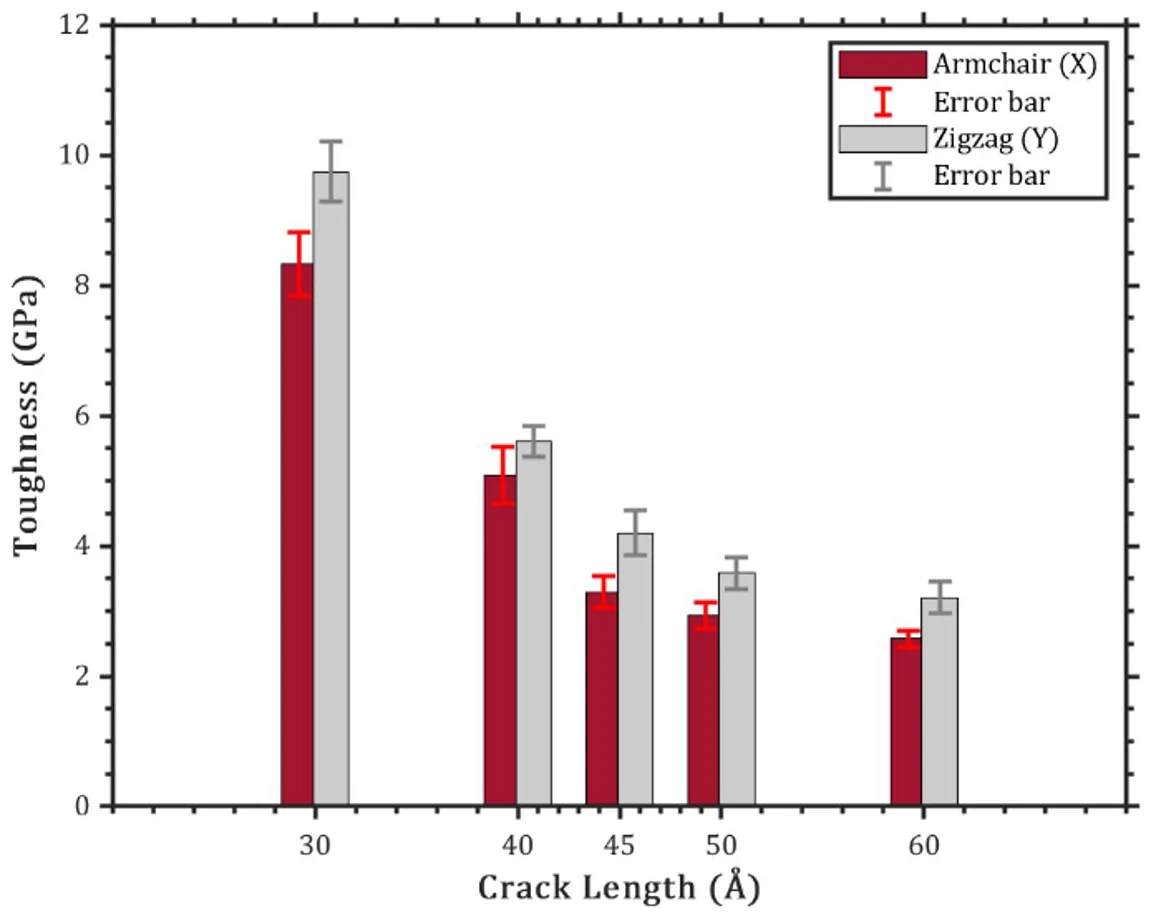
Variation of toughness of TNS with crack length at 300K.

Fig 11 illustrates the variation of toughness of the TNS as a function of crack length ranging from 30 Å to 60 Å, at a temperature of 300 K and with a 90° central crack orientation. The data reveals a clear decreasing trend in toughness with increasing crack length, for both the X and Y directions. In the X direction, toughness drops from 8.33 GPa at 30 Å to 2.57 GPa at 60 Å, representing a 69.1% reduction. Similarly, in the Y direction, the toughness decreases from 9.75 GPa to 3.21 GPa, corresponding to a 67.1% reduction. Although both directions experience significant degradation, the nanosheet exhibits consistently higher toughness in the Y direction across all crack lengths, highlighting the relatively better damage tolerance along the Y orientation. The substantial reduction in toughness with crack extension reflects the critical role of crack propagation in weakening the structural energy absorption capacity of the nanosheet. The superior fracture toughness in the Y-direction, despite its lower elastic modulus, reveals important insights into the energy dissipation mechanisms within the TNS structure. Fracture toughness represents the material’s ability to absorb energy during crack propagation, which depends not only on bond strength but also on the availability of energy dissipation pathways. In the Y-direction, the tortuous stress path through the zigzag trigonal network provides multiple opportunities for energy dissipation through: (1) elastic deformation of the flexible trigonal rings, (2) activation of secondary slip systems that redistribute stress, and (3) crack tip blunting mechanisms that reduce stress concentrations. The trigonal geometry creates a natural “crack tip shielding” effect where stress is redistributed through the ring structures rather than concentrating at a single point. Conversely, the X-direction’s higher stiffness comes at the cost of brittleness, as the more direct stress transfer pathway provides fewer opportunities for energy dissipation before catastrophic failure. The temperature-dependent reduction in fracture toughness (70–74% decrease from 200K to 1000K) indicates that thermal activation disrupts these energy dissipation mechanisms, possibly by reducing the effectiveness of trigonal ring flexibility and promoting more direct bond-breaking processes.

**Fig 12:**
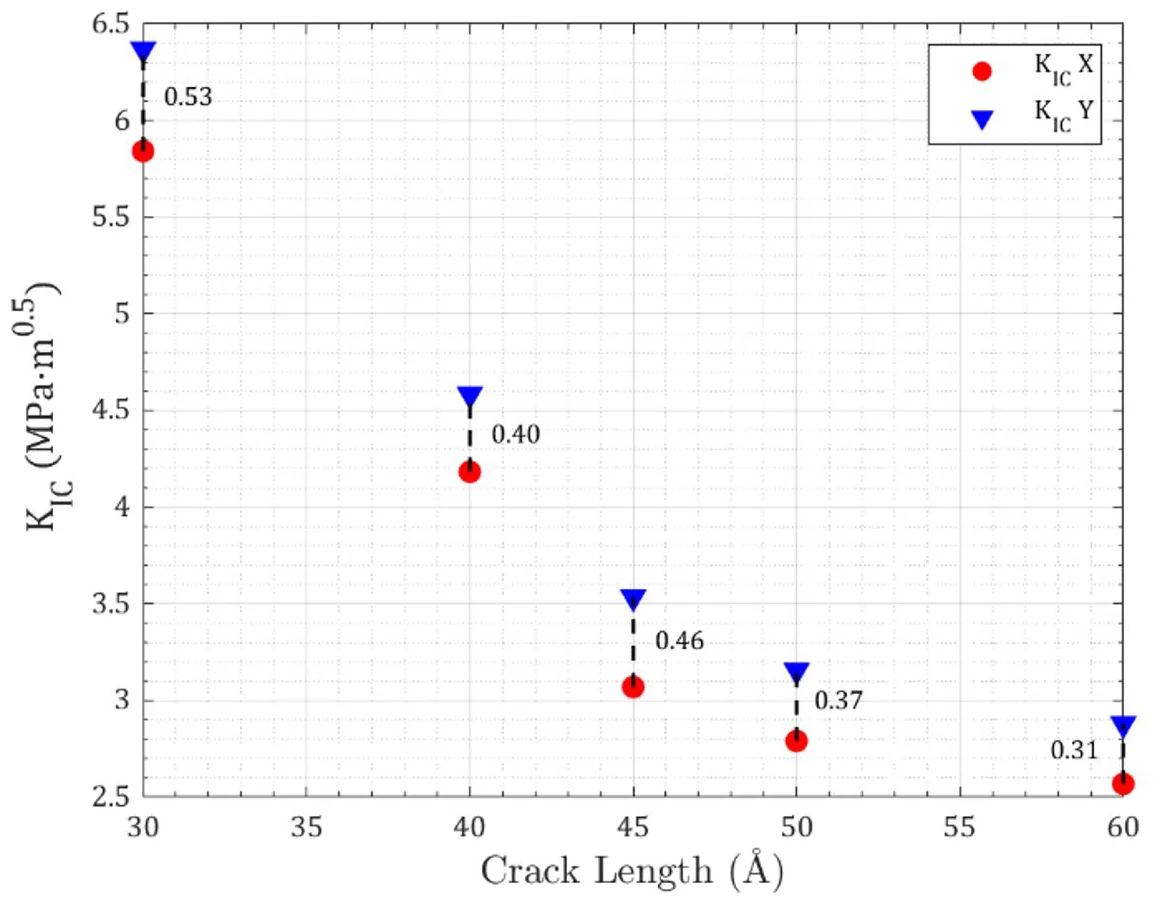
Variation of stress intensity factor of TNS with crack length at 300K.

Fig 12 illustrates the variation of the critical stress intensity factor ${\text{K}}_{\text{Ic}}$of a TNS with respect to crack length (in Å) for both the X and Y directions. As the crack length increases from 30 Å to 60 Å, a noticeable decrease in ${\text{K}}_{\text{Ic}}$ is observed, indicating a degradation in the material’s resistance to crack propagation. In the X direction, ${\text{K}}_{\text{Ic}}$ reduces from 5.84 $\text{MPa}\cdot \sqrt{\text{m}}$ at 30 Å to 2.57 $\text{MPa}\cdot \sqrt{\text{m}}$ at 60 Å, corresponding to a reduction of approximately 56%. In the Y direction, the decrease is from 6.37 to 2.88, which reflects a 55% drop. These closely matched percentage reductions suggest that although the initial toughness is higher in the Y direction, the rate of deterioration due to crack extension is similarly severe in both orientations. This trend underscores the dominant role of crack length in reducing fracture resistance, regardless of directional anisotropy.

The systematic degradation of mechanical properties with increasing crack length, shown in Figs 9-12, must be interpreted in the context of the finite system size. For the longest crack studied (60 Å), the crack length-to-width ratio (2a/2h) is 0.30, which remains within the range where LEFM assumptions are reasonably valid for finite-width specimens [39]. Beyond this ratio, crack-boundary interactions would become increasingly significant, and the stress intensity factor formula used in Eq. (1) would require additional correction factors. Therefore, our conclusions regarding crack length effects are confined to the range 30–60 Å, which is appropriate for the system dimensions employed. It is worth noting that this range of crack lengths relative to system size is comparable to those used in recent MD studies of fracture in other 2D carbon allotropes [8,16,40], facilitating direct comparison of results.

In the present study, TNS exhibited $K_{Ic}$ values ranging from 2.57 to 7.08 $\text{MPa}\sqrt{\text{m}}$ at 300 K, depending on loading direction, crack orientation, and crack length. The upper end of this range exceeds the experimentally measured fracture toughness of pristine graphene ($K_{Ic}\approx \text{4}.\text{0}\text{ MPa}\sqrt{\text{m}}$ [47]), indicating that under optimal conditions, TNS can offer greater crack propagation resistance despite its significantly lower elastic modulus (210–215 GPa vs. ~ 1 TPa for graphene). This enhanced toughness is attributed to unique energy dissipation mechanisms enabled by the trigonal ring topology, including crack tip shielding and stress redistribution through the distorted bond network.

Comparative studies reveal that other 2D materials exhibit substantially lower fracture toughness. Buckled hexagonal materials such as silicene, AlN, and SiC possess $K_{Ic}$ values of 0.8–2.2 $\text{MPa}\sqrt{\text{m}}$ [48], while most buckled 2D hexagonal sheets exhibit $K_{Ic}\leq \text{0}.\text{8}\text{ MPa}\sqrt{\text{m}}$ [49]. γ-graphyne and boron nitride nanosheets show values of 2.21–2.62 $\text{MPa}\sqrt{\text{m}}$ [50], decreasing with temperature, a trend consistent with our thermal softening observations. Hexagonal boron nitride (h-BN) displays direction-dependent toughness (3.94–4.68 $\text{MPa}\sqrt{\text{m}}$ [51]), closely matching the anisotropic range of TNS. Single crystal silicon, a benchmark brittle material, exhibits $K_{Ic}\approx \text{1}.\text{0}\text{ MPa}\sqrt{\text{m}}$ [52], meaning even the lower bound of TNS toughness (2.57 $\text{MPa}\sqrt{\text{m}}$) remains ~2.6 times higher.

**Fig 13:**
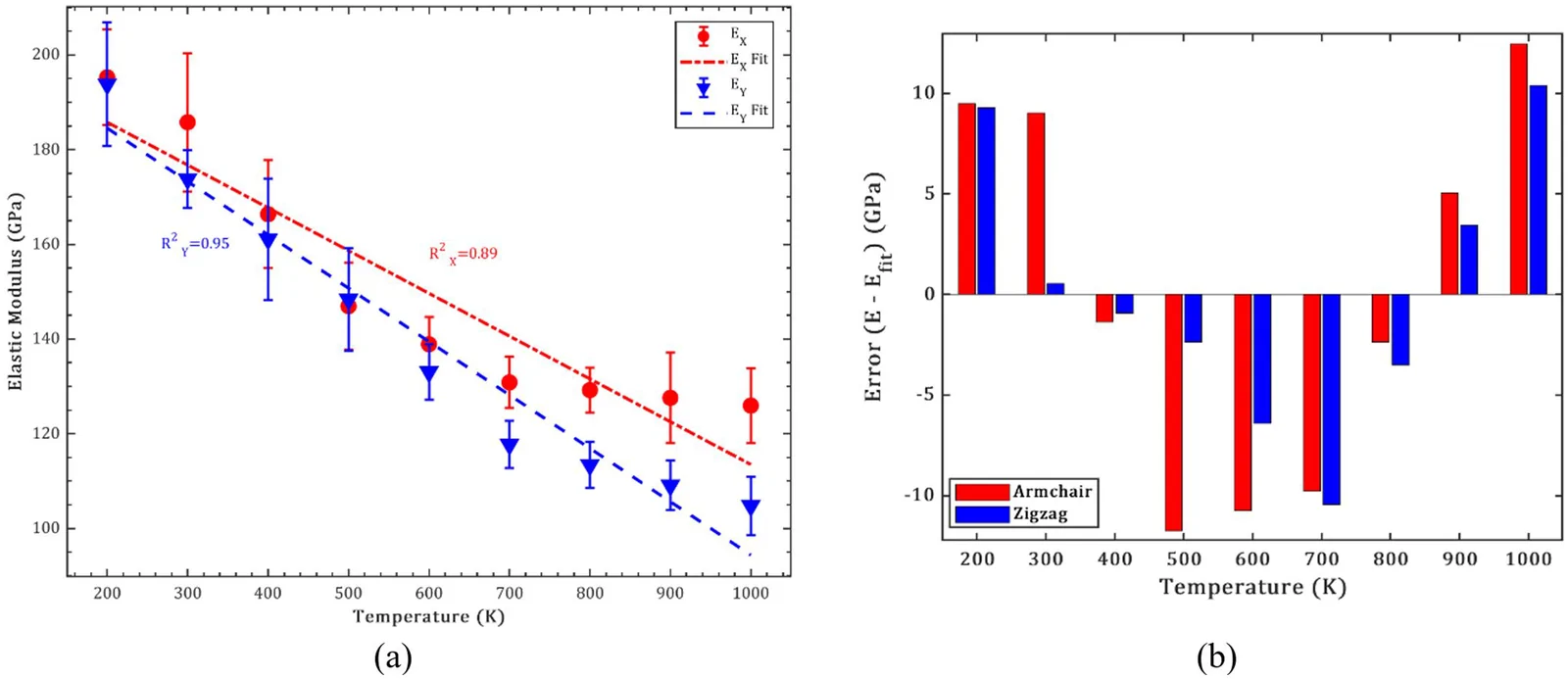
Temperature-dependent (a) variation of elastic modulus for TNS (b) elastic modulus fitting error.

Fig 13 illustrates the variation of the elastic modulus of a TNS with a central crack of 40 Å length oriented at a 90° angle, positioned approximately at the center of a square nanosheet with side length 203.23 Å. The results are presented for both X and Y directions under a temperature range of 200 K to 1000 K. As temperature increases, a clear declining trend is observed in the elastic modulus for both directions, indicating thermal softening of the material. In the X-direction, the elastic modulus drops from 195.26 GPa at 200 K to 125.95 GPa at 1000 K, while in the Y-direction, the decline is more pronounced, falling from 193.81 GPa to 104.78 GPa over the same temperature range. This anisotropic reduction may be attributed to directional dependence of bond weakening and enhanced atomic vibrations near the crack tip, which are exacerbated under elevated thermal agitation. The presence of the crack not only reduces the overall stiffness but also amplifies the temperature sensitivity of the mechanical response, especially in the zigzag orientation due to its distinct lattice configuration and stress concentration behavior. The Wachtman equation, originally proposed to describe the temperature dependence of elastic modulus, is employed in the form: $\text{E}(\text{T})={\text{E}}_{\text{0}}-{\text{E}}_{\text{1}}\text{T }{\text{e}}^{-\frac{{\text{T}}_{\text{o}}}{\text{T}}}$ [53]. To model the thermal softening behavior of materials. In the present study, this relation is used to fit the elastic modulus data as a function of temperature. For the armchair direction, the fitted model is given by: ${\text{E}}_{\text{X}}(\text{T})=\text{203}.\text{9}-\text{0}.\text{09T }{\text{e}}^{-\frac{\text{0}.\text{01}}{\text{T}}}$ with a coefficient of determination ${\text{R}}^{\text{2}}=\text{0}.\text{89}$, while for the zigzag direction, the corresponding fit is: ${\text{E}}_{\text{Y}}(\text{T})=\text{207}.\text{1}-\text{0}.\text{11T }{\text{e}}^{-\frac{\text{0}.\text{01}}{\text{T}}}$ with ${\text{R}}^{\text{2}}=\text{0}.\text{95}$, indicating a strong agreement between the fitted model and the numerical data. These results confirm the suitability of the Wachtman-type equation in capturing the nonlinear temperature dependence of elastic modulus, particularly in anisotropic materials such as 2D nanosheets.

The pronounced thermal softening observed in TNS with increasing temperature stems from the complex interplay between thermal activation of atomic vibrations and the pre-existing structural strain within the trigonal network. At elevated temperatures, increased thermal energy enhances atomic mobility and weakens interatomic bonds through thermal expansion, reducing the effective bond strength and stiffness. The distorted bond angles (60° and 150°) in TNS create internal stress concentrations that are particularly sensitive to thermal activation, as these strained bonds require less additional energy to overcome binding forces compared to the relaxed bonds in pristine graphene. The Wachtman-type temperature dependence captures the non-linear thermal softening behavior, where the exponential term reflects the thermally-activated nature of bond weakening processes. The greater temperature sensitivity in the Y-direction indicates that the zigzag-oriented trigonal bonds are more susceptible to thermal degradation, possibly due to their higher internal strain energy state. Additionally, thermal fluctuations preferentially activate slip systems and defect migration pathways that are aligned with the zigzag direction, accelerating the degradation of mechanical properties through thermally-assisted crack tip processes.

**Fig 14:**
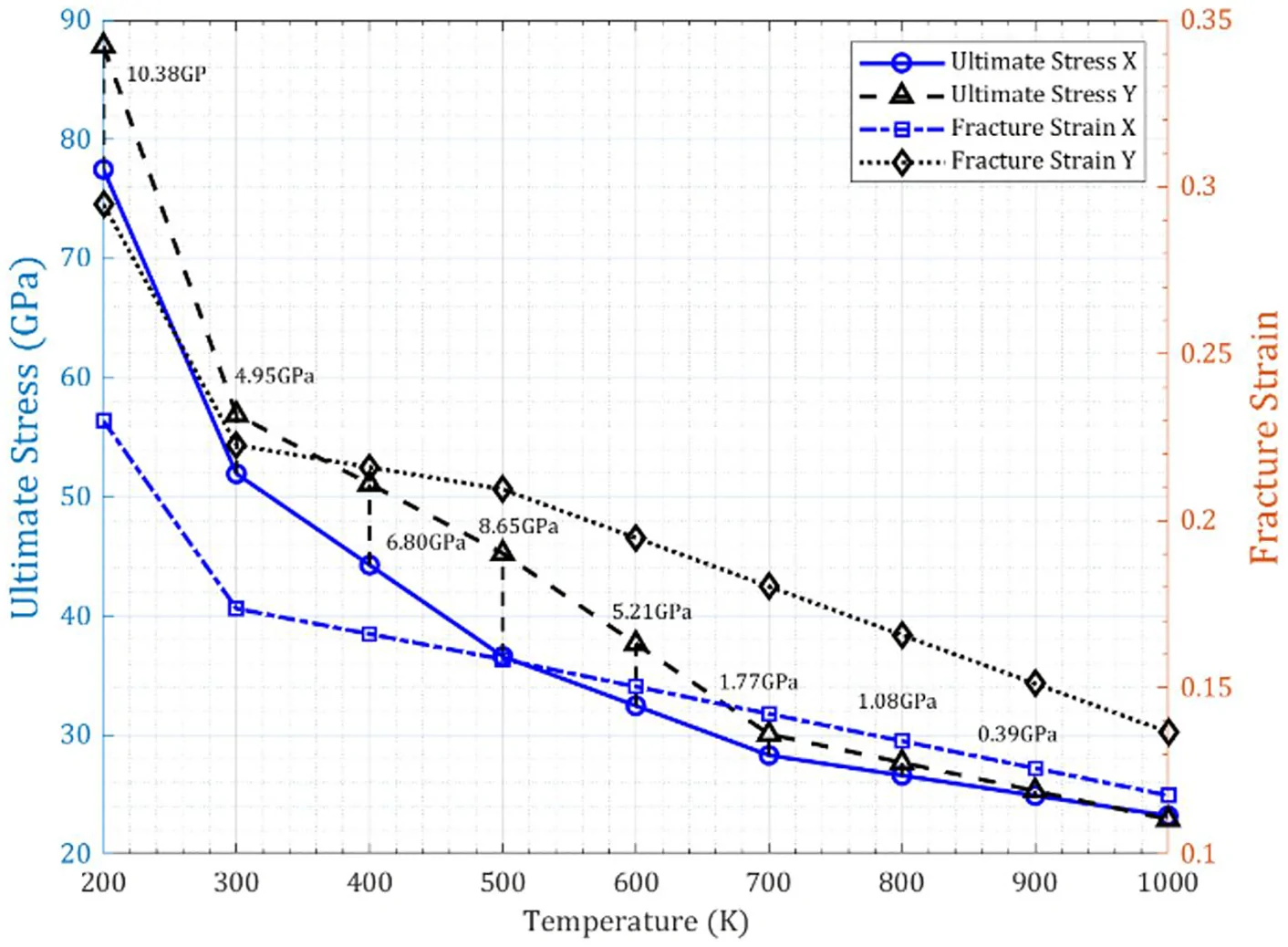
Temperature-dependent variation of ultimate stress and fracture strain of TNS.

Fig 14 demonstrates the temperature-dependent variation of ultimate stress and fracture strain for a TNS. The data are presented for both the X and Y directions over a temperature range from 200 K to 1000 K. As the temperature increases, both ultimate stress and fracture strain exhibit a noticeable decrease, indicating a thermal softening behavior of the material. The percentage variation in ultimate stress from 200 K to 1000 K is −70.02% for the X direction and −73.85% for the Y direction, with the Y direction showing a greater reduction. Similarly, the fracture strain decreases by −48.73% in the X direction and −53.66% in the Y direction, indicating a loss of ductility as temperature increases. These percentage variations highlight the significant influence of temperature on the material’s mechanical properties, with both strength and strain capacity diminishing as the temperature rises. The greater reduction in the Y direction could be attributed to the specific atomic arrangement and bonding characteristics along the Y orientation, which may be more sensitive to thermal degradation.

**Fig 15:**
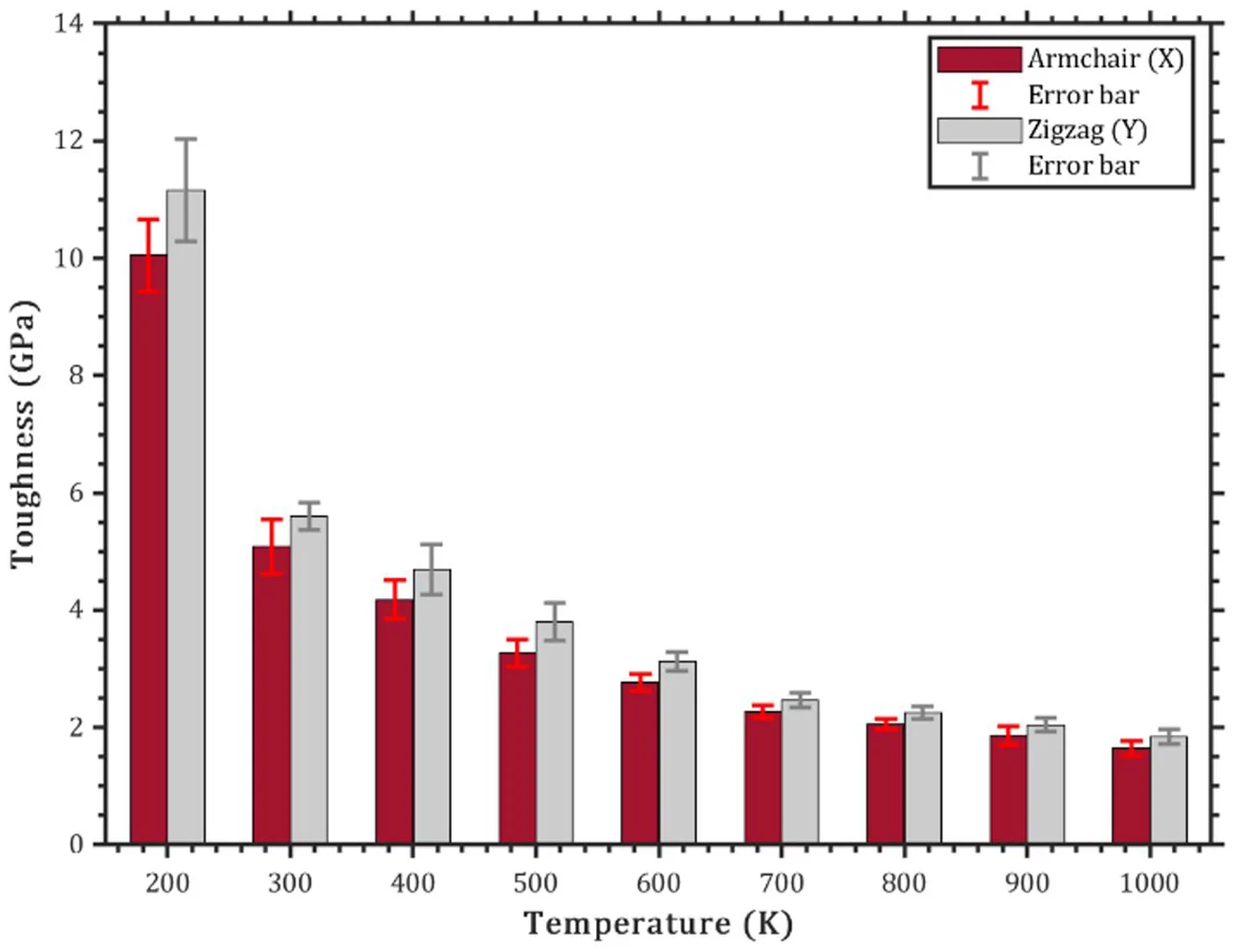
Temperature-dependent variation of toughness for TNS.

Fig 15 illustrates the temperature-dependent variation of toughness for a TNS. The toughness values are shown for both the X and Y directions across a temperature range from 200 K to 1000 K. As the temperature increases, both directions exhibit a noticeable decrease in toughness, reflecting a material softening behavior and a reduction in its resistance to crack propagation at elevated temperatures. In the X direction, the toughness decreases from 10.05 GPa at 200 K to 1.65 GPa at 1000 K, representing a −83.64% reduction. Similarly, in the Y direction, toughness drops from 11.16 GPa at 200 K to 1.84 GPa at 1000 K, showing a −83.54% decrease. The Y direction maintains a higher toughness across the temperature range compared to the X direction, which could be due to the difference in atomic bonding characteristics and the anisotropic nature of the material. The observed decrease in toughness in both directions is consistent with the thermal softening phenomenon, where the atomic vibrations and thermal expansion reduce the material’s structural integrity and crack resistance. The higher initial toughness in the Y direction suggests it is more resistant to crack growth compared to the X direction, but both directions exhibit a significant loss in toughness as the temperature increases, ultimately reducing the material’s fracture resistance at higher temperatures.

**Fig 16:**
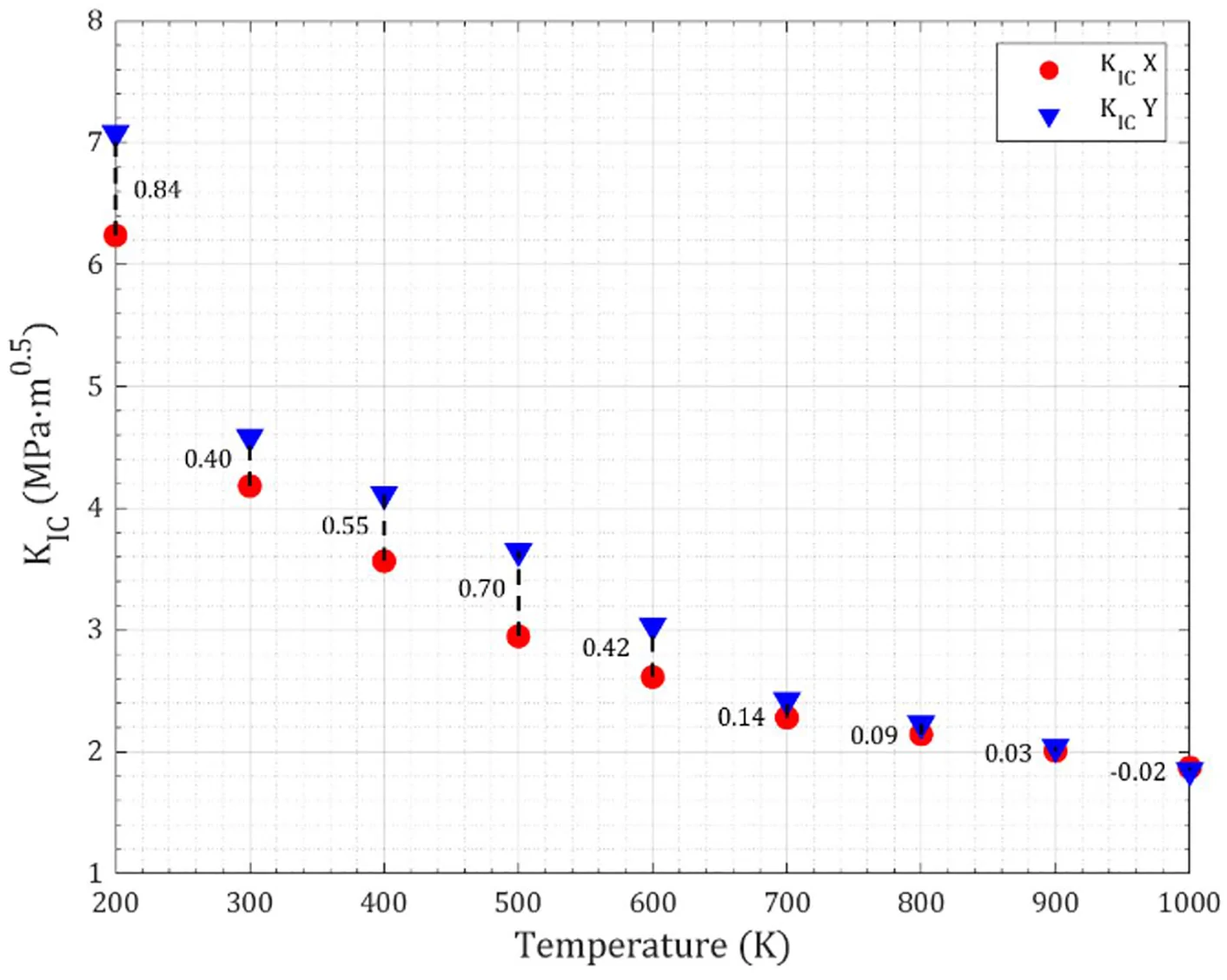
Temperature-dependent variation of stress intensity factor of TNS.

Fig 16 presents the temperature-dependent variation of the ${\text{K}}_{\text{Ic}}$ for a TNS with a central crack of 40 Å length, oriented at a 90° angle, within a nanosheet of side length 203.23 Å. The data are shown for both the X and Y directions across a temperature range from 200 K to 1000 K. As temperature increases, the ${\text{K}}_{\text{Ic}}$ in both directions exhibits a substantial decrease, indicating a reduction in the material’s resistance to crack propagation under thermal stress. In the X direction, ${\text{K}}_{\text{Ic}}$ decreases from 6.24 $\text{MPa}\cdot \sqrt{\text{m}}$ at 200 K to 1.87 $\text{MPa}\cdot \sqrt{\text{m}}$ at 1000 K, representing a −70.04% reduction. Similarly, in the Y direction, ${\text{K}}_{\text{Ic}}$ drops from 7.08 $\text{MPa}\cdot \sqrt{\text{m}}$ at 200 K to 1.85 $\text{MPa}\cdot \sqrt{\text{m}}$ at 1000 K, showing a −73.86% decrease. The Y direction maintains a higher ${\text{K}}_{\text{Ic}}$ at all temperatures compared to the X direction, indicating a greater resistance to crack propagation along the Y direction. The percentage variation for both directions reflects the thermal softening and weakening of the material, as higher temperatures lead to increased atomic vibrations and enhanced thermal expansion, reducing the overall fracture toughness. The X direction shows a more significant decrease in ${\text{K}}_{\text{Ic}}$. A sensitivity analysis suggests that the ${\text{K}}_{\text{Ic}}$ is more sensitive to temperature changes in the Y direction, as the Y orientation experiences a more substantial reduction in fracture resistance compared to the X direction. As temperature increases, the atomic bonds in the Y direction are more prone to thermal degradation, thus exhibiting a steeper decline in ${\text{K}}_{\text{Ic}}$. Both directions demonstrate significant weakening, with the Y direction being slightly more vulnerable to thermal softening at elevated temperatures.

Table 2 presents the critical energy release rate (${\text{G}}_{\text{c}}$) for TNS as functions of crack angle, crack length, and temperature, calculated using the relationship ${\text{G}}_{\text{c}}={\text{K}}_{\text{c}}^{\text{2}}/\text{E}$. This parameter quantifies the material’s resistance to crack propagation in terms of energy dissipation, which is of direct practical relevance for assessing structural reliability.

The data presented in Table 2 reveal several important trends in the fracture resistance of TNS. With respect to crack angle dependence, $G_{c}$ decreases monotonically as the crack angle increases from 0° to 90° for both loading directions. The armchair direction exhibits a reduction from 148.0 J/m² at 0° to 94.2 J/m² at 90°, representing a 36% decrease, while the zigzag direction shows a more pronounced reduction from 201.1 J/m² to 120.9 J/m², corresponding to a 40% decrease. Notably, the zigzag direction consistently maintains higher energy release rates across all crack angles, indicating superior fracture resistance compared to the armchair orientation.

Regarding crack length effects, the critical energy release rate demonstrates severe degradation as crack length increases. For the armchair direction, $G_{c}$ drops from 159.8 J/m² at a crack length of 30 Å to 45.2 J/m² at 60 Å, a dramatic reduction of approximately 72%. Similarly, the zigzag direction experiences a decrease from 205.6 J/m² to 64.7 J/m² over the same crack length range, corresponding to a 69% reduction. This substantial weakening underscores the critical role of crack size in determining the structural integrity of TNS, with longer cracks creating more severe stress concentrations that facilitate easier crack propagation.

The temperature dependence of $G_{c}$ reveals significant thermal softening effects. As temperature increases from 200 K to 1000 K, the critical energy release rate in the armchair direction declines from 199.4 J/m² to 27.8 J/m², an 86% reduction. The zigzag direction exhibits a similar trend, decreasing from 258.4 J/m² at 200 K to 32.6 J/m² at 1000 K, also representing an 86% decrease. Although the zigzag direction maintains higher $G_{c}$ values throughout the entire temperature range, both directions experience comparable relative degradation at elevated temperatures. This strong temperature sensitivity highlights the importance of considering thermal conditions when evaluating the fracture resistance of TNS-based devices.

## 4. Conclusions

In this study, the fracture behavior of TNS with pre-existing central cracks was comprehensively examined using NEMD simulations. The key findings and contributions of this work are summarized below:

- **First systematic investigation of fracture in defective trigraphene:** A comprehensive database of mechanical properties for cracked TNS was established for the first time, including elastic modulus, ultimate tensile strength, fracture strain, toughness, stress intensity factor, and critical energy release rate as functions of crack angle (0°–90°), crack length (30–60 Å), and temperature (200–1000 K).
- **Discovery of direction-dependent failure modes:** Under armchair (X) loading, symmetric mode-I brittle fracture with high stress concentration at crack tips was observed, consistent with classical fracture mechanics. In contrast, under zigzag (Y) loading, a previously unreported crack deflection mechanism was revealed, wherein fracture paths deviated toward sheet boundaries due to diffuse stress fields and crack tip shielding, a direct consequence of TNS’s unique trigonal topology with 60° and 150° bond angles.
- **Inverse anisotropic mechanical behavior:** Contrary to conventional trends in graphene, TNS was found to exhibit higher elastic modulus in the X-direction but superior ultimate strength, fracture strain, toughness, and fracture resistance in the Y-direction. This inverse anisotropy was attributed to the distorted bond network of trigraphene, which creates different load transfer pathways depending on loading direction.
- **New predictive models for crack-angle dependence:** Trigonometric functions ($E(\theta )=E_{\text{0}}+E_{\text{1}}\text{cos}(\text{2}\theta )+E_{\text{2}}\text{cos}(\text{4}\theta )$) were derived and validated to accurately capture the variation of elastic modulus with crack orientation. The fitted coefficients ($E_{\text{1}}=\text{9}.\text{3}$ for X-direction, $E_{\text{1}}=\text{1}\text{2}.\text{4}$ for Y-direction) quantified the greater sensitivity of the zigzag direction to crack orientation.
- **Power-law degradation models for crack length effects:** The reduction in mechanical properties with increasing crack length was shown to follow a power-law relationship ($E=E_{\text{0}}(\text{1}-a/h{)}^{n}$) with exponents n=1.52 (X) and n=1.67 (Y), providing quantitative tools for predicting stiffness loss in cracked TNS structures.
- **First quantification of critical energy release rate for defective TNS:** The critical energy release rate was computed as $G_{c}=K_{c}^{\text{2}}/E$ for all simulated conditions. It was observed that $G_{c}$ decreased by 36–40% as crack angle increased from 0° to 90°, by 69–72% as crack length increased from 30 Å to 60 Å, and by 86% as temperature increased from 200 K to 1000 K. These values provide essential input parameters for continuum-level fracture models.
- **Thermal sensitivity exceeding other 2D carbon allotropes:** Pronounced thermal softening was observed in TNS, with toughness reduced by over 80% from 200 K to 1000 K, a higher thermal sensitivity than graphene or other carbon allotropes. This behavior was attributed to the inherent lattice strain in TNS’s trigonal structure, which makes it more susceptible to thermal activation of bond weakening.

The dataset established in this study was intended to provide essential input parameters (cohesive zone properties, fracture toughness, energy release rates) for higher-scale models. Building on recent advances in machine-learning interatomic potentials [45], future work could focus on developing MLIPs specifically tailored to TNS. Such potentials, trained on comprehensive DFT datasets encompassing strained, defective, and thermally excited configurations, would enable more accurate predictions of fracture behavior while maintaining computational efficiency. This approach would also facilitate direct validation of the present results and provide a robust foundation for multiscale modeling frameworks that bridge atomistic fracture mechanisms to continuum-scale predictions of TNS-based devices. Additionally, future studies could address crack deflection under mixed-mode and cyclic loading conditions, systematically examine the effects of trigon density and edge chirality on fracture behavior, and derive multiscale models grounded in the atomistic simulations presented herein.

## Supporting information

S1 Data Numerical data underlying the quantitative figures. Numerical values underlying the mechanical-property and fracture-mechanics results presented in Figs 5–16, including the effects of crack angle, crack length, and temperature on elastic modulus, ultimate stress, fracture strain, toughness, and critical stress intensity factor.
(XLSX)
